# Modeling the association between psychological capital and nurses’ job performance: The moderating role of intolerance of uncertainty

**DOI:** 10.1371/journal.pone.0350761

**Published:** 2026-06-17

**Authors:** Seyedeh Melika Kharghani Moghadam, Ehsan Taheri, Ezzat Jafarjalal, Hossein Ebrahimi

**Affiliations:** 1 Nursing and Midwifery Care Research Center, Health Management Research Institute, Department of Health Services, School of Public Health, Iran University of Medical Sciences, Tehran, Iran; 2 Department of Clinical Psychology, School of Medicine, Shahid Beheshti University of Medical Sciences, Tehran, Iran; 3 Nursing and Midwifery Care Research Center, Health Management Research Institute, School of Nursing and Midwifery, Iran University of Medical Sciences, Tehran, Iran; 4 Nursing and Midwifery Care Research Center, Health Management Research Institute, Department of Occupational Health and Safety Engineering, School of Public Health, Iran University of Medical Sciences, Tehran, Iran; University of Luzon, PHILIPPINES

## Abstract

This cross-sectional analytical study aimed to examine the relationship between psychological capital (PsyCap) and nurses’ job performance, as well as the moderating role of intolerance of uncertainty (IU). A total of 212 nurses working in hospitals affiliated with Iran University of Medical Sciences participated. Verbal informed consent was obtained from all participants, as approved by the Ethics Committee of Iran University of Medical Sciences (IR.IUMS.REC.1401.561). Data collection tools included standardized questionnaires on PsyCap, IU, and job performance. Data were analyzed using structural equation modeling (SEM) with SmartPLS software. The measurement model was assessed for reliability and validity, and the structural model was tested for the study hypotheses. The findings indicated that PsyCap was positively and significantly associated with nurses’ job performance (β = 0.38, p < 0.001). IU was not significantly associated with job performance. However, IU negatively moderated the PsyCap–performance relationship (β = −0.16, p = 0.005). These findings suggest that PsyCap may be positively related to nurses’ job performance, although its positive effects may be attenuated under high IU. Interventions aimed at strengthening PsyCap alongside uncertainty-management strategies may help optimize performance in clinical settings.

## 1. Introduction

Nurses constitute the largest professional group within healthcare systems and play a critical role in ensuring patient safety and quality of care. Their job performance—defined as the effectiveness and efficiency with which professional duties are carried out—has direct implications for clinical outcomes, patient satisfaction, and healthcare system sustainability [1]. Globally, performance-related failures and medical errors remain a major concern, contributing to an estimated 2.6 million preventable deaths annually [2]. In Iran, empirical evidence suggests that a substantial proportion of nurses experience performance challenges associated with high workload, psychological strain, and limited organizational support [3]. These concerns underscore the need to identify modifiable psychological and organizational factors that may be associated with nurses’ job performance.

Within this context, psychological capital (PsyCap) has emerged as a key positive psychological resource for improving work-related outcomes. PsyCap is a state-like construct comprising self-efficacy, hope, resilience, and optimism [4–6]. Extensive research across occupational settings has demonstrated that higher levels of PsyCap are associated with improved well-being, job satisfaction, and performance [7–10]. In nursing populations specifically, both international and Iranian studies consistently report positive associations between PsyCap and indicators of job performance and adaptive functioning [11,12]. These findings suggest that PsyCap represents a valuable personal resource that enables nurses to cope more effectively with job demands and perform optimally under pressure.

However, healthcare environments are inherently characterized by uncertainty, ambiguity, and rapid decision-making, particularly in hospital settings. Intolerance of uncertainty (IU)—defined as a dispositional tendency to perceive uncertain situations as threatening— has been shown to be associated with psychological distress, anxiety, and impaired decision-making among healthcare professionals [13,14]. Evidence from Iranian nursing samples indicates that IU levels are particularly elevated in high-acuity units such as emergency departments and intensive care units [15]. Despite growing recognition of IU as an important vulnerability factor, its role in shaping job performance outcomes has received limited empirical attention.

Importantly, existing research has largely examined PsyCap as a direct predictor of job performance, with little consideration of contextual or psychological boundary conditions that may strengthen or weaken its effects. This represents a critical gap in the literature. Drawing on stress and coping theories [14] and positive organizational behavior frameworks [5], it is plausible that high levels of IU may undermine the effective mobilization of PsyCap resources in uncertain clinical environments. In other words, while PsyCap may generally be associated with better performance, its positive impact may be attenuated when nurses experience high IU. Addressing this gap, the present study explicitly examines IU as a moderating variable in the relationship between PsyCap and nurses’ job performance.

To empirically test this framework, we employed structural equation modeling (SEM), which allows for the simultaneous evaluation of measurement validity and hypothesized structural relationships among latent constructs [16,17]. SEM is particularly well-suited for examining complex psychosocial models involving moderation effects and has been increasingly applied in nursing and health sciences research [17].

**Fig 1:**
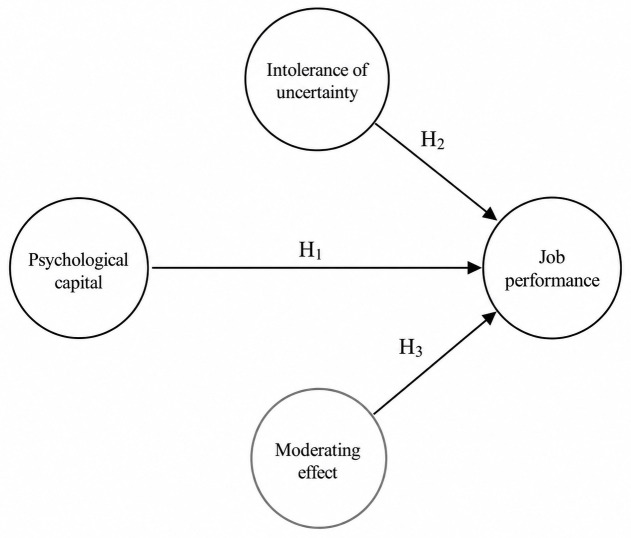
Proposed conceptual model (PLS-SEM framework).

Accordingly, the present study aimed to examine the relationship between PsyCap and nurses’ job performance, while also investigating the role of IU as a potential moderating factor. Based on positive organizational behavior theory and stress–coping perspectives, we hypothesized that PsyCap would be positively associated with nurses’ job performance (H1), that IU would be negatively associated with job performance (H2), and that IU would moderate the relationship between PsyCap and job performance, such that the positive effect of PsyCap would be weaker at higher levels of IU (H3). Fig 1 illustrates the proposed conceptual model and hypothesized relationships.

## 2. Materials and methods

### 2.1. Study population

This research was a cross-sectional descriptive-analytical study conducted on nurses employed in hospitals affiliated with Iran University of Medical Sciences. The adequacy of the sample size in this study was assessed based on both methodological guidelines for SEM and power analysis. According to Kline (2023), a minimum of 10 participants per estimated parameter is recommended [17]. Given our model with approximately 18 free parameters (including factor loadings, error variances, structural paths, and latent variances), the minimum required sample size was about 180 participants. Similarly, Hair et al. (2021) recommend at least 200 cases for complex SEM models [16]. Therefore, a minimum sample size of 200 participants was considered to evaluate the relationships in the model.

After eligibility screening, a simple random sampling method was used to select participants from the personnel rosters of hospitals affiliated with Iran University of Medical Sciences. To minimize selection bias, recruitment was conducted across different work shifts, and participation was voluntary. In total, 235 nurses were invited to participate, of whom 212 completed the survey (response rate = 90.2%).

### 2.2. Data collection tools

In this study, data were collected using the Luthans Psychological Capital Questionnaire, Intolerance of Uncertainty Scale (IUS), and a Job Performance Questionnaire. Given that all data were collected using self-report questionnaires at a single time point, several procedural strategies were employed to reduce the risk of common method bias. These included ensuring participant anonymity, emphasizing that there were no right or wrong answers, and using standardized and validated instruments. Despite these precautions, the possibility of common method bias cannot be completely ruled out.

#### 2.2.1. Luthans Psychological Capital Questionnaire (2007).

This questionnaire contains 24 items and measures four components: hope, resilience, optimism, and self-efficacy. The items are scored on a six-point Likert scale to assess PsyCap. The Cronbach’s alpha coefficient for this questionnaire has been reported to be above 0.7 [18].

#### 2.2.2. Intolerance of Uncertainty Scale (IUS).

This questionnaire was developed by Freeston et al. (1994) to measure individuals’ tolerance for uncertain and ambiguous situations. It consists of 27 items scored on a five-point Likert scale. This scale distinguishes between two factors in differentiating anxious individuals from healthy ones: 1) self-referential implications and negative behaviors associated with uncertainty, and 2) the perception that uncertainty is unfair and spoils everything. The Cronbach’s alpha coefficient for this scale has been reported as 0.94 [19–21]. The 27-item version of the Intolerance of Uncertainty Scale (IUS) was used in this study to provide a more comprehensive assessment of the construct, capturing both self-referential and behavioral responses to uncertainty. This version has demonstrated strong psychometric properties in Persian-speaking populations and was selected to ensure greater construct coverage for the moderation analysis.

#### 2.2.3. Job performance questionnaire.

This questionnaire was developed by Patterson (1970) and includes 15 items. The questions were scored on a five-point Likert scale [22]. The Cronbach’s alpha coefficient for this questionnaire has been reported as 0.78 [23].

### 2.3. Instrument validity in Iran

To document local validity, we cited Iranian psychometric evidence for all instruments. The Persian PCQ-24 has shown a hierarchical four-factor structure with excellent fit (CFI ≈ 0.99, RMSEA≈0.03), Cronbach’s α across subscales ranging 0.85–0.89, and 4-week test–retest r ≈ 0.80 [24]. The Persian IUS-12 demonstrated a stable two-factor structure (prospective and inhibitory anxiety) with CR ≈ 0.86 and Cronbach’s α ≈ 0.89, alongside acceptable content validity indices. The Persian Paterson Job Performance Questionnaire has been widely used in Iranian nursing and non-nursing samples, with internal consistency typically α ≈ 0.85–0.90 and split-half reliability around 0.85 [25]. These findings support the adequacy of the instruments for use in the Iranian context.

### 2.4. Methodology

To conduct the study, ethical approval was obtained from the Ethics Committee of Iran University of Medical Sciences (IR.IUMS.REC.1401.561). Verbal informed consent was obtained from all participants prior to data collection, as approved by the ethics committee. After participant selection, the study purpose was explained and verbal informed consent was obtained. Questionnaires were distributed on 9/11/2024 and collected on 19/11/2024. Data were entered into SPSS v22 for cleaning and descriptive analyses and then analyzed in SmartPLS for SEM. Given that the primary aim of this study was theory-driven hypothesis testing, the model was specified to focus on the hypothesized relationships among the core constructs. Demographic variables were therefore not included as control variables in the structural model to preserve theoretical clarity and model parsimony.

SEM was used to examine interrelated relationships among the study variables. This study utilized two aspects of the SEM approach: measurement model and structural model. Confirmatory factor analysis (CFA) was applied within the measurement model to determine the contribution of each item (or observed variable) in measuring the latent construct; The measurement model evaluated the link between observed and latent variables. The structural model, on the other hand, evaluates the relationships between latent variables [26].

The widespread application and popularity of this technique among researchers in health and medical sciences are due to its ability to provide a quantitative method for theory testing while addressing the complexities of analyzing variable relationships in human studies [27]. Unlike traditional linear models, such as multiple regression, SEM can also estimate measurement errors [28].

## 3. Results

In this study, a random sample of 212 eligible nurses was analyzed. Most participants were female (70.8%) and the average age and work experience were 38.76 ± 10.66 years and 14.53 ± 9.58 years, respectively. This demographic profile reflects a mid-career nursing workforce, supporting the applicability of findings to clinical settings where experienced staff predominate.

Table 1 presents scores for job performance, PsyCap and IU and their dimensions. PsyCap showed a relatively high overall mean (103.43 ± 15.87), with the highest subscale mean for self-efficacy and the lowest for optimism; IU showed its two facets (F1, F2) at moderate levels. Higher self-efficacy suggests a strong belief in capability among nurses, whereas comparatively lower optimism indicates a potential target for interventions (e.g., strength-based coaching) aimed at sustaining positive expectancies and performance.

**Table 1:** Mean scores for job performance, psychological capital (PsyCap), and intolerance of uncertainty (IU) (N = 212).

Variable	Mean	SD	Dimensions	Mean	SD
**Job performance**	62.27	9.86			
**Psychological capital (PsyCap)**	103.43	15.87	Self-efficacy	27.38	5.3
			Hope	26.33	5.75
			Resilience	26.13	4.05
			Optimism	23.58	3.74
**Intolerance of uncertainty (IU)**	78.21	2.78	F1	42.86	9.73
			F2	35.35	8.02

Note. Values are means ± standard deviations.

F1 = self-referential and behavioral implications of uncertainty;

F2 = perceptions that uncertainty is unfair and spoils everything.

After determining the scores for the factors under investigation within the target population, the correlation coefficients between these factors were calculated. Table 2 reports correlations. Job performance correlated moderately with PsyCap (r = 0.45, p < 0.001) but not with IU (r = 0.09, p = 0.185). The moderate association indicates that nurses with greater psychological resources tend to perform better. The absence of a direct link between IU and performance suggests that IU influences performance mainly as a moderator.

**Table 2:** Correlation coefficients for job performance, psychological capital (PsyCap), and intolerance of uncertainty (IU) (N = 212).

Variable	Job Performance	Psychological Capital (PsyCap)	Intolerance of Uncertainty (IU)
Job Performance	–	0.45***	0.09
Psychological Capital (PsyCap)	–	–	−0.07
Intolerance of Uncertainty (IU)	–	–	–

**Note.** Values are Pearson’s correlation coefficients (two-tailed).

***p < 0.001.

These correlation results indicate a significant positive association between PsyCap and job performance, providing preliminary support for H1, while IU was not significantly correlated with job performance, offering no support for H2 at the bivariate level.

**Fig 2:**
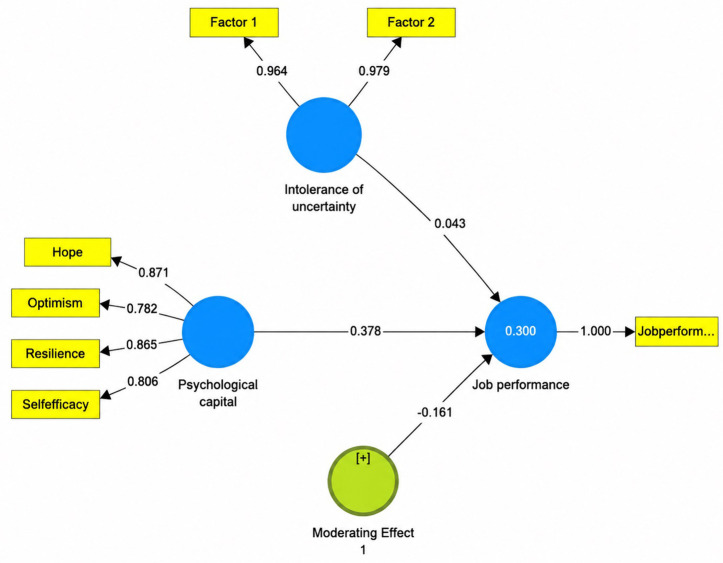
Structural equation model illustrating the relationships among psychological capital (PsyCap), intolerance of uncertainty (IU), and job performance (N = 212).

Following the assessment of correlation coefficients among the examined variables, the proposed conceptual model was tested using Smart PLS 3 software. Fig 2 depicts the implemented conceptual model in the PLS software, encompassing both the measurement model and the structural model.

The PLS-SEM **measurement model** showed acceptable psychometrics (**Table 3**): all standardized loadings exceeded conventional thresholds (≥0.70 for most indicators), composite reliability was high for PsyCap (CR = 0.90) and IU (CR = 0.97), and AVE values were ≥0.50 (PsyCap AVE = 0.69; IU AVE = 0.94). These indices support convergent validity and internal consistency, indicating that latent constructs are measured reliably and warrant structural inference.

**Table 3:** Measurement model results for psychological capital (PsyCap) and intolerance of uncertainty (IU) (N = 212).

	Loading	T statistic	Composite reliability	AVE
**Psychological capital (PsyCap)**			0.90	0.69
**Self-efficacy**	0.80	19.47		
**Hope**	0.87	36.61		
**Resilience**	0.86	24.72		
**Optimism**	0.78	14.30		
**Intolerance of uncertainty (IU)**			0.97	0.94
F1*	0.96	8.49		
F2**	0.97	7.98		

* Indecisiveness has implications of self-referentiality and negative behavior.

** Indecision is unfair and spoils everything.

The structural model results (Table 4; Fig 2) indicated a significant positive path from PsyCap to job performance (β = 0.38, T = 4.81, p < 0.001), a non-significant direct path from IU to job performance (β = 0.04, T = 0.67, p = 0.503), and a significant negative moderating role of IU on the PsyCap→performance relationship (β = −0.16, T = 2.81, p = 0.005). The PsyCap coefficient reflects a moderate positive effect—practically, higher levels of PsyCap were associated with better job performance. The non-significant IU→performance path suggests that uncertainty tolerance does not depress performance on average; rather, IU dampens the benefits of PsyCap—i.e., at higher levels of IU, the performance payoff from PsyCap is smaller. This implies that initiatives to reduce IU (e.g., uncertainty-management training, clear protocols) may restore or amplify the performance gains from PsyCap-building programs.

**Table 4:** Structural model results for the hypothesized relationships (N = 212).

Hypothesized relationships	Coefficient (β)	T-statistic	P-value
**H1: Psychological capital (**PsyCap**) → Job performance**	0.38	4.81	p < 0.001
**H**_**2**_ **: Intolerance of uncertainty (IU)→ Job performance**	0.04	0.67	0.503
**H**_**3**_ **: moderating role/ moderating relationship→ Job performance**	−0.16	2.81	0.005

Structural equation modeling results indicated a significant positive association between PsyCap and job performance, thus supporting H1, whereas the direct effect of IU on job performance was not significant and H2 was not supported. Importantly, the interaction between PsyCap and IU was significant and negative, indicating that IU moderated the PsyCap–job performance relationship in support of H3. Overall, the model explained a moderate proportion of variance in job performance (R² = 0.24). Effect size analysis showed a medium effect of PsyCap (f² = 0.18), a negligible effect of IU (f² = 0.01), and a small-to-moderate effect of the interaction term (f² = 0.06).

Model predictive relevance, assessed by cross-validated redundancy, was Q² = 0.24. This value indicates medium predictive relevance for job performance (cf. heuristic cut-offs ≈0.02 small, 0.15 medium, 0.35 large), suggesting the model explains practically useful variance in performance and can inform program design.

## 4. Discussion

The findings of the present study indicate a significant positive association between psychological capital (PsyCap) and nurses’ job performance, whereas intolerance of uncertainty (IU) was not significantly associated with job performance at the direct level. In addition, IU was found to significantly moderate the relationship between PsyCap and job performance, such that the strength of this association was weaker at higher levels of IU. These findings provide a comprehensive picture of how individual psychological resources and uncertainty-related vulnerability are related to nurses’ performance outcomes.

From an empirical perspective, the positive association between PsyCap and job performance underscores the importance of internal psychological resources in nursing practice. PsyCap encompasses self-efficacy, hope, resilience, and optimism—capabilities that enable nurses to remain motivated, adaptable, and effective when faced with heavy workloads and emotional demands. The observed PsyCap–performance relationship is consistent with prior empirical studies conducted in diverse healthcare settings, including hospital-based nursing samples in Iran and other countries, which have reported similar positive effects of PsyCap on work-related outcomes [29–33].

However, evidence in the literature regarding the strength of the PsyCap–performance relationship is not entirely consistent across contexts. For example, studies conducted in highly protocolized clinical environments, such as intensive care units and emergency departments in European and East Asian healthcare systems, have reported weaker or non-significant associations once contextual factors such as job autonomy, leadership climate, or work engagement were taken into account [34,35]. These discrepancies suggest that the relationship between PsyCap and job performance may vary depending on situational characteristics, including the degree of discretion nurses have in their roles and the level of environmental uncertainty they experience.

In line with this context-sensitive interpretation, the moderating role of IU observed in the present study provides important insight into when PsyCap is most likely to translate into improved performance. Although IU was not directly associated with job performance, its interaction with PsyCap indicates that nurses who struggle to tolerate uncertainty may find it more difficult to mobilize their psychological resources effectively. Previous research conducted in hospital and primary care settings has shown that high IU is more strongly related to anxiety, stress, and emotional exhaustion than to performance per se, particularly in environments characterized by ambiguous guidelines or rapidly changing clinical conditions [36–39]. Our findings extend this literature by demonstrating that IU can act as a boundary condition that constrains the performance-enhancing effects of PsyCap.

The present findings can be further interpreted within the framework of established nursing performance theories, particularly the Job Demands–Resources (JD–R) model. According to the JD–R framework, job performance results from the balance between job demands (e.g., workload, time pressure, emotional strain) and job and personal resources (e.g., psychological capital, support, autonomy). Within this framework, PsyCap can be conceptualized as a personal resource that enhances motivation and resilience, whereas IU represents a psychological vulnerability that amplifies perceived job demands under uncertain conditions. When IU is high, uncertainty may be appraised as threatening, increasing cognitive and emotional demands and thereby weakening the positive motivational pathway through which PsyCap supports performance. By integrating PsyCap and IU within the JD–R model, this study provides a more nuanced, theory-driven explanation of nurses’ job performance in uncertainty-laden clinical environments.

From a practical perspective, the findings of this study suggest several considerations for healthcare organizations and nursing management, particularly in relation to supporting nurses’ performance in complex and uncertainty-laden clinical environments.

First, the observed association between psychological capital (PsyCap) and job performance indicates that interventions aimed at strengthening PsyCap components—such as self-efficacy, hope, resilience, and optimism—may be beneficial. These interventions could be incorporated into continuing professional development programs and training initiatives designed to enhance nurses’ psychological resources.

Second, the moderating role of intolerance of uncertainty (IU) suggests that efforts to better manage uncertainty in clinical settings may help support the effective utilization of psychological resources. Strategies such as improving clarity of clinical protocols, enhancing communication processes, and providing access to decision-support tools may contribute to more structured and predictable working environments.

Third, organizational and leadership practices that promote supportive supervision and psychological safety may facilitate more adaptive responses to uncertainty. Encouraging open communication, collaborative problem-solving, and timely feedback may help nurses navigate ambiguous situations more effectively.

Finally, broader organizational factors, including staffing adequacy, workload management, and structured shift systems, may also play a role in shaping the context in which psychological resources are expressed. Attention to these factors may contribute to more stable and supportive work environments, which in turn may be associated with improved performance outcomes.

Overall, these considerations highlight the potential value of integrated approaches that address both individual psychological resources and organizational conditions when aiming to support nurses’ job performance.

## 5. Conclusion

The findings of this study emphasize the importance of psychological capital (PsyCap) as a critical personal resource for supporting nurses’ job performance, while also drawing attention to the constraining role of IU in complex clinical environments. Rather than reiterating numerical results, this study highlights the practical relevance of considering both psychological resources and uncertainty-related challenges when designing strategies to support nursing performance.

From a practice and management perspective, the results suggest that improving nurses’ job performance requires integrated approaches that go beyond isolated training initiatives. Interventions aimed at strengthening PsyCap—such as enhancing self-efficacy, resilience, hope, and optimism—should be complemented by organizational efforts to reduce uncertainty and improve nurses’ capacity to tolerate ambiguity. At the policy level, incorporating competencies related to psychological capital and uncertainty management into nursing education standards, leadership development programs, and workforce planning guidelines may contribute to more resilient healthcare systems and safer patient care.

Most importantly, this study provides a model describing how IU moderates the relationship between psychological capital and nurses’ job performance. By articulating this moderating mechanism, the study advances existing performance research beyond simple main-effect models and offers a more nuanced framework for future research, management practice, and policy development in uncertainty-laden healthcare settings.

### 5.1. Limitations and future research

This study has several limitations that should be acknowledged. First, its cross-sectional design restricts the ability to infer causal relationships between PsyCap, IU, and job performance; longitudinal or experimental designs are therefore needed to establish temporal ordering and causal mechanisms.

Second, reliance on self-report questionnaires raises the possibility of common method variance and social desirability bias, which may have influenced some of the observed associations. Additionally, as all variables were measured using self-report instruments collected at a single time point, the findings may be subject to common method bias. Although procedural remedies were applied, such as assuring anonymity and using validated measures, future studies should consider multi-source or longitudinal data to further reduce this concern.

Third, the sample was drawn from nurses employed in hospitals affiliated with a single university in Tehran. As these hospitals operate within a specific cultural, organizational, and healthcare context, the findings should be interpreted with caution when generalizing to other regions, countries, or healthcare systems with different professional norms, resource constraints, and uncertainty structures. Fourth, unmeasured contextual factors—such as leadership style, workload intensity, or organizational culture—may also influence job performance and could partially confound the observed relationships.

In addition to addressing these limitations, several directions for future research are suggested. First, longitudinal studies could examine how changes in PsyCap and IU over time predict subsequent changes in job performance, providing stronger evidence regarding temporal relationships. Intervention-based designs may operationalize PsyCap development through structured training programs targeting self-efficacy, hope, resilience, and optimism, while IU-focused interventions could assess the effects of uncertainty-management training, decision-support tools, or coping-skills workshops on reducing IU levels.

Second, future research should incorporate broader geographic and organizational samples, including hospitals from different regions of Iran as well as international healthcare settings, to enhance generalizability and capture potential cultural or systemic differences. Finally, the integration of multi-source and longitudinal data—such as supervisor ratings, patient safety indicators, or objective performance metrics—would allow for a more comprehensive and robust examination of how psychological resources and uncertainty tolerance jointly influence nurses’ job performance.

## Supporting information

S1 File Sampling procedure details. (DOCX)
