# Supplementary material for: Modeling the association between psychological capital and nurses’ job performance: The moderating role of intolerance of uncertainty
Source: PLoS One. 2026 Jun 17;21(6):e0350761. doi: 10.1371/journal.pone.0350761 (PMC13274869; doi:10.1371/journal.pone.0350761)
Supplement: S1 File — (DOCX) [file pone.0350761.s001.docx]

**Supplementary Appendix A. Sampling Procedure Details**

A simple random sampling procedure was implemented using SPSS software (Select Cases → Random Sample) based on combined personnel rosters obtained from hospital human resources units. To account for potential nonresponse, approximately 10% oversampling was applied when generating the initial randomized list. Recruitment followed the pre-generated random order across morning, evening, and night shifts. No convenience substitutions were permitted.
