# Supplementary material for: Modeling the association between psychological capital and nurses’ job performance: The moderating role of intolerance of uncertainty
Source: PLoS One. 2026 Jun 17;21(6):e0350761. doi: 10.1371/journal.pone.0350761 (PMC13274869; doi:10.1371/journal.pone.0350761)
Supplement: Attachment — Submitted filename: Our reply to reviewers.docx [file pone.0350761.s003.docx]

**Dear Editor**

We would like to express our sincere gratitude to the Editor for the time and effort dedicated to handling our manuscript. We have carefully considered all the reviewers’ comments and the journal’s requirements, and we have thoroughly revised the manuscript accordingly. We believe that these revisions have significantly improved the clarity, rigor, and overall quality of the paper, and we hope that the revised version will now be suitable for publication in PLOS ONE.

**Response to Reviewers**

**Reviewer 1:**

The manuscript is well-structured and scientifically solid; however, some sections need tightening for clarity, consistency, and stronger alignment with PLOS ONE’s reporting standards.

Please ensure consistent terminology for constructs (e.g., “psychological capital” vs. “PsyCap”) and for consent type (“verbal” vs. “written”) across all sections.

**Response:** We thank the reviewer for the positive evaluation of the manuscript and for this helpful comment. In response, we carefully revised the manuscript to improve clarity and consistency in terminology in line with PLOS ONE reporting standards. Specifically, we standardized the use of terminology for key constructs by consistently using psychological capital (PsyCap) at first mention and PsyCap thereafter throughout the manuscript. In addition, the type of informed consent has been consistently reported as verbal informed consent across all relevant sections (Abstract, Methods, and Ethics statement). These revisions have enhanced clarity and ensured consistency throughout the manuscript.

***Abstract***

1. The first sentence could explicitly state the design (e.g., “a cross-sectional analytical study”) for methodological clarity.

**Response:** The first sentence of the Abstract was revised to explicitly state the study design as a cross-sectional analytical study to improve methodological clarity.

2. Consider shortening the last two sentences to emphasize implications more succinctly.

**Response:** The final two sentences of the Abstract were condensed to more succinctly emphasize the key practical implications of the findings.

3. Ensure that all abbreviations (e.g., SEM, IU) are defined at first mention.
**Response:** All abbreviations, including intolerance of uncertainty (IU) and structural equation modeling (SEM), are now defined at first mention in the Abstract.

***Introduction***

1. The introduction is comprehensive, but the flow can be improved by reducing background repetition on PsyCap.

**Response:** We thank the reviewer for this valuable suggestion. To improve the flow of the Introduction, we reduced repetitive background information on psychological capital by consolidating its definition and empirical evidence into a single, concise paragraph. This revision preserves the theoretical foundation while improving readability and narrative coherence.

2. The research gap could be highlighted earlier (around line 80–85) to strengthen reader engagement.

**Response:** In response to this comment, we revised the Introduction to explicitly highlight the research gap at an earlier stage. Specifically, we emphasized the lack of studies examining intolerance of uncertainty as a moderating condition in the relationship between psychological capital and nurses’ job performance, thereby strengthening the rationale and engagement for the present study.

3. Figure 1 should be referenced with a more explicit description in text (e.g., “Figure 1 illustrates the proposed moderating framework…”).

**Response:** We have revised the manuscript to include a clearer and more explicit in-text reference to Figure 1. The text now explicitly states that Figure 1 illustrates the proposed conceptual and moderating framework linking psychological capital, intolerance of uncertainty, and nurses’ job performance.

***Methods***

1. The section is detailed and clear. However, the ethics paragraph still inconsistently refers to written consent in the ethics statement while “verbal consent” is noted in the main text. This should be reconciled to match ethics committee approval.

**Response:** We thank the reviewer for pointing out this inconsistency. We carefully revised the ethics paragraph to ensure full consistency across the manuscript. All references to informed consent now clearly and consistently state that verbal informed consent was obtained from all participants, in accordance with the approval of the ethics committee.

2. The sampling procedure description is excellent, but please move some technical detail (SPSS commands, oversampling explanation) to a supplementary appendix for brevity.

**Response:** We thank the reviewer for the positive evaluation of the sampling procedure. In response to this comment, we streamlined the sampling description in the Methods section for brevity and clarity, while moving technical details (e.g., SPSS commands and oversampling procedures) to a supplementary appendix.

3. Clarify whether any control variables (e.g., gender, age, experience) were tested in the SEM model.

**Response:** We clarified this point in the Methods section. As the primary aim of the study was theory-driven hypothesis testing, the structural model was specified to focus on the hypothesized relationships among the core constructs. To preserve theoretical clarity and model parsimony, demographic variables were not included as control variables in the SEM.

4. Add brief justification for using the 27-item IUS rather than the IUS-12 short form, since the latter is more common in nursing research.

Response: We added a brief justification in the Methods section for using the 27-item version of the Intolerance of Uncertainty Scale. The IUS-27 was selected to provide more comprehensive construct coverage and due to its strong psychometric validation in Persian samples, which was particularly important for the moderation analysis.

***Results***

1. Figures and tables are informative but need uniform style (same decimal precision and table notes).

**Response:** We thank the reviewer for this helpful comment. All tables were carefully revised to ensure a uniform presentation style throughout the Results section. Specifically, decimal precision was standardized across all numerical values, p-values were reported consistently (e.g., p < 0.001), and table notes were harmonized using a single, consistent format in line with PLOS ONE reporting standards.

2. Provide sample size (N=212) in every table caption.

**Response:** In response to this comment, the sample size (N = 212) has been added to the captions of all tables to improve clarity and transparency.

3. The interpretation sentences following Tables 2 and 4 could more clearly relate numeric values to the study hypotheses (e.g., “Thus, H1 was supported, while H2 was not…”).

**Response:** We revised the interpretation sentences following Tables 2 and 4 to explicitly link the reported statistical results to the study hypotheses. The Results section now clearly states which hypotheses were supported and which were not, based on the numerical findings.

4. Report R² and f² values for main paths to support the discussion on effect size and model strength.

**Response:** In response to this comment, we added R² and f² values to the Results section to provide additional information on model explanatory power and effect sizes of the main and interaction paths. These additions strengthen the interpretation of model robustness and effect magnitude.

***Discussion***

1. The discussion is strong but should better distinguish between empirical findings and interpretive commentary—currently they blend in several paragraphs.

**Response:** We thank the reviewer for this constructive comment. The Discussion section was substantially revised to clearly distinguish between empirical findings and interpretive commentary. Specifically, we separated the neutral summary of the main results from their theoretical interpretation and comparison with prior studies, thereby improving clarity, structure, and narrative flow.

2. When citing conflicting evidence, specify study contexts (e.g., countries, healthcare settings) to enrich the comparative analysis.

**Response:** In response to this comment, we revised the Discussion to explicitly specify study contexts when discussing conflicting evidence. The revised text now highlights differences in countries, healthcare settings, and clinical environments to provide a more nuanced and context-sensitive comparative analysis.

3. Add a short paragraph linking findings to existing nursing performance theories or models (e.g., Job Demands–Resources framework).

**Response:** We added a new paragraph linking the study findings to established nursing performance theories, specifically the Job Demands–Resources (JD–R) framework. This addition situates psychological capital and intolerance of uncertainty within a theory-driven model of job performance, strengthening the conceptual contribution of the study.

4. The “Practical Implications” subsection is excellent but could be condensed to highlight 3–4 priority recommendations.

**Response:** The Practical Implications subsection was revised and condensed to highlight four priority recommendations. These focused recommendations emphasize psychological capital development, uncertainty management, supportive leadership practices, and organizational strategies to enhance nurses’ job performance.

***Conclusion***

1. Rephrase to avoid restating numerical findings; focus on implications for practice, management, and policy.

**Response:** We thank the reviewer for this helpful suggestion. The Conclusion section was revised to avoid restating numerical or statistical findings and to place greater emphasis on implications for clinical practice, management, and policy. The revised text now highlights the broader relevance of the findings for nursing performance improvement and healthcare system development.

2. The final sentence could more directly state the contribution: “This study provides an empirically validated model explaining how intolerance of uncertainty moderates the effect of psychological capital on nurses’ performance.”

**Response:** We revised the final sentence of the Conclusion to more explicitly state the study’s contribution. The Conclusion now clearly emphasizes that this study provides an empirically validated model explaining how intolerance of uncertainty moderates the effect of psychological capital on nurses’ job performance.

***Limitations and Future Research***

1. Well-written, but should add a note about the cultural context (Iranian hospitals) as a possible boundary to generalization.

**Response:** We thank the reviewer for this valuable suggestion. We revised the Limitations section to explicitly acknowledge the cultural and organizational context of Iranian hospitals as a potential boundary condition for generalizing the findings to other regions and healthcare systems.

2. Suggest specifying how longitudinal or intervention designs might operationalize PsyCap or IU changes.

**Response:** In response to this comment, we expanded the Future Research section to specify how longitudinal and intervention-based designs could operationalize changes in psychological capital and intolerance of uncertainty. The revised text now outlines potential PsyCap development and uncertainty-management interventions and highlights their value for clarifying causal pathways.

***References***

1. Check consistency of et al. usage (journal requires up to 6 authors before “et al.”).

**Response:** We revised the reference list to ensure consistent use of “et al.” in accordance with the journal’s guidelines, listing up to six authors before using “et al.” where applicable.

2. Ensure uniform citation style (e.g., spacing before volume and issue numbers).

**Response:** The reference list was carefully reviewed and edited to ensure a uniform citation style throughout, including consistent spacing, punctuation, and formatting of volume and issue numbers, in line with PLOS ONE requirements.

3. Verify the accuracy of Iranian journal references (some may lack DOIs).

**Response:** We verified the accuracy of all references, with particular attention to Iranian journal articles. For references without DOIs, complete and accurate bibliographic information has been provided in accordance with the journal’s citation guidelines.

***Language and Style***

1. The manuscript is generally fluent but occasionally verbose; shorten sentences over 25 words.

**Response:** We revised the manuscript to improve conciseness by shortening long sentences and reducing unnecessary wording across sections, while preserving meaning and scientific clarity.

2. Ensure consistent tense use—Methods and Results in past tense; Introduction and Discussion in present tense where applicable.

**Response:** We standardized verb tense throughout the manuscript. Methods and Results were edited to consistently use past tense, and Introduction and Discussion were revised to use present tense where appropriate, particularly for interpretation and general statements.

3. Avoid redundancy in transitions (e.g., remove phrases like “it should be noted that…”).

**Response:** We edited transitional phrases to eliminate redundancy and improve readability, removing unnecessary filler expressions and streamlining paragraph openings and linking sentences.
