# Supplementary material for: Modeling the association between psychological capital and nurses’ job performance: The moderating role of intolerance of uncertainty
Source: PLoS One. 2026 Jun 17;21(6):e0350761. doi: 10.1371/journal.pone.0350761 (PMC13274869; doi:10.1371/journal.pone.0350761)
Supplement: Attachment — Submitted filename: Our_reply_to_reviewers_auresp_2.docx [file pone.0350761.s004.docx]

**Dear Editor,**

We thank you for your careful evaluation of our revised manuscript and for the constructive comments provided. We appreciate the opportunity to further revise and improve the manuscript.

In preparing this revision, we have carefully addressed all points raised in your editorial assessment. The manuscript has been reviewed in detail to ensure clarity, internal consistency, and alignment with the reporting standards of PLOS ONE. In particular, we have ensured consistency in ethical reporting and informed consent documentation across all sections and submission materials, revised the wording throughout the manuscript to avoid causal interpretations in accordance with the cross-sectional design, and strengthened methodological transparency by explicitly addressing common method bias.

All changes have been incorporated into the revised manuscript, and a detailed point-by-point response is provided below to indicate how each comment has been addressed.

We hope that the revised manuscript meets the journal’s requirements and is suitable for publication.

**Additional Editor Comments:**

1- Thank you for submitting the revised version of the manuscript. The revised paper demonstrates substantial improvement, reflecting significant effort to address the concerns raised during the previous review. The manuscript is now better organized, with strengthened analytical reporting, and key sections such as the discussion, conclusion, and limitations are more focused and aligned with the study’s objectives. Notably, the clarification of the study design, the more explicit reporting of structural equation modeling results, and the clearer articulation of the study’s theoretical and practical relevance are commendable.
However, following careful editorial assessment, the manuscript is not yet ready for acceptance in its current form. Although the remaining issues do not warrant rejection, they are sufficiently significant to require further revision before a final decision can be made. Most notably, there is an inconsistency in the ethics and consent documentation. The manuscript reports verbal informed consent, while other submission materials indicate written consent. This inconsistency must be corrected thoroughly and consistently across the manuscript, ethics statement, and all submission metadata. Additionally, the ethics approval should clearly support the form of consent that was actually obtained, including any waiver of written consent if applicable.
There are also unresolved concerns regarding the reporting and justification of the intolerance of uncertainty instrument. The manuscript utilizes the 27-item measure, yet some justification relies on discussion of the shorter IUS-12, which may cause confusion. The rationale, validation support, and interpretation should consistently correspond to the instrument actually administered. The reference list must also be reviewed carefully, as inclusion of any retracted source is unacceptable and such citations must be removed or replaced with valid references.
**Response:** We sincerely thank the Editor for identifying this important inconsistency in the ethics and consent documentation. We have carefully reviewed the manuscript and the submission materials and confirm that verbal informed consent was obtained from all participants. The previous statement indicating that participants signed a written consent form was an error in wording in the Ethics Statement and did not reflect the actual consent procedure used in the study.

To correct this issue, we have revised the Ethics Statement and all relevant parts of the manuscript to consistently state that verbal informed consent was obtained. We also clarified that the use of verbal informed consent, including the waiver of written informed consent, was approved by the Ethics Committee of Iran University of Medical Sciences under the ethics code IR.IUMS.REC.1401.561.

The revised Ethics Statement now reads:

“This study was conducted with the approval of the Ethics Committee of Iran University of Medical Sciences under the ethics code IR.IUMS.REC.1401.561. Prior to data collection, the study objectives were explained to all participants, and verbal informed consent was obtained voluntarily from each participant without any coercion. The use of verbal informed consent and the waiver of written informed consent were approved by the Ethics Committee. All participant information was kept strictly confidential by the researchers.”

We have also reviewed the submission metadata and accompanying documents to ensure consistency regarding the consent procedure.

2- Although the discussion section has improved, some passages still contain wording that may be interpreted as causal or overly prescriptive. As the study is cross-sectional and based on self-report data collected at a single time point, interpretations must remain associative rather than causal throughout the manuscript. Statements suggesting that one construct improves, determines, or directly leads to another should be revised. Furthermore, the manuscript would benefit from a clearer statement regarding common method bias. Even if no formal statistical test was conducted, any procedural steps taken to reduce this concern should be briefly explained, and the issue should be acknowledged transparently in the limitations.

Response: We sincerely thank the Editor for this important and insightful comment. We fully agree that, given the cross-sectional design and reliance on self-report data collected at a single time point, all interpretations should remain strictly associative rather than causal.

In response, we conducted a comprehensive and systematic revision of the manuscript to eliminate potentially causal language. Specifically, terms such as “effect,” “impact,” “influence,” and “improves” were carefully reviewed and replaced with more appropriate expressions such as “association,” “relationship,” and “is related to” throughout the Abstract, Results, Discussion, and Conclusion sections. This revision ensures full consistency with the observational nature of the study.

In addition, we have further clarified the interpretation of findings in the Discussion section by explicitly framing the results in terms of associations rather than causal mechanisms. The Conclusion has also been revised to avoid prescriptive or causal implications

Regarding common method bias, we have now addressed this issue explicitly. A statement has been added to the Methods section describing procedural steps taken to reduce this bias, including ensuring participant anonymity, emphasizing voluntary participation, and using standardized and validated instruments. Furthermore, the Limitations section has been expanded to transparently acknowledge the potential influence of common method bias due to the use of self-report data collected at a single time point.

We believe that these revisions have substantially improved the interpretive rigor and methodological transparency of the manuscript, and we hope that they fully address the Editor’s concern.

3- The manuscript is therefore invited for further revision. In preparing the next version, all remaining concerns should be addressed carefully, accurately, and consistently throughout the full manuscript and submission materials. The authors are encouraged to review the revised paper line by line to ensure that no conflicting statements remain and that all reporting is fully aligned with journal standards. A clear and detailed point-by-point response should also be provided, explaining exactly how each concern has been addressed.

**Response:** We sincerely thank the Editor for inviting us to revise the manuscript further and for providing clear guidance for the next revision. We have carefully reviewed the entire manuscript line by line to ensure that all remaining concerns have been addressed accurately and consistently.

In particular, we checked the manuscript and submission materials for consistency in ethical reporting and informed consent documentation. All references to informed consent have now been revised to consistently indicate that verbal informed consent was obtained, as approved by the Ethics Committee of Iran University of Medical Sciences, and that the requirement for written informed consent was waived.

We also systematically reviewed the manuscript to ensure that the interpretation of findings remains appropriate for a cross-sectional, self-report study. Potentially causal or overly prescriptive wording was revised throughout the Abstract, Results, Discussion, Conclusion, and Practical Implications sections.

In addition, we added a clearer statement regarding common method bias in the Methods section and expanded the Limitations section to acknowledge this issue transparently.

A detailed point-by-point response has been provided below, explaining exactly how each concern has been addressed. We hope that the revised manuscript and accompanying response letter now fully meet the journal’s reporting standards.
