# Supplementary material for: Modeling the association between psychological capital and nurses’ job performance: The moderating role of intolerance of uncertainty
Source: PLoS One. 2026 Jun 17;21(6):e0350761. doi: 10.1371/journal.pone.0350761 (PMC13274869; doi:10.1371/journal.pone.0350761)
Supplement: Peer review history

# Peer review: Decision Letter 0

**DOI:** 10.1371/journal.pone.0350761.r001

**Authors:** Abdelaziz Hendy

6 Aug 2025

PONE-D-25-35558

Modeling the Effect of Psychological Capital on Nurses' Job Performance with the Moderating Role of Intolerance of Uncertainty

PLOS ONE

Dear Dr. Hossein Ebrahimi,

Thank you for submitting your manuscript to PLOS ONE. After careful consideration, we feel that it has merit but does not fully meet PLOS ONE’s publication criteria as it currently stands. Therefore, we invite you to submit a revised version of the manuscript that addresses the points raised during the review process.

Please submit your revised manuscript by  Sep 20 2025 11:59PM. If you will need more time than this to complete your revisions, please reply to this message or contact the journal office at plosone@plos.org. When you're ready to submit your revision, log on to https://www.editorialmanager.com/pone/ and select the 'Submissions Needing Revision' folder to locate your manuscript file.

Please include the following items when submitting your revised manuscript:

- A rebuttal letter that responds to each point raised by the academic editor and reviewer(s). You should upload this letter as a separate file labeled 'Response to Reviewers'.
- A marked-up copy of your manuscript that highlights changes made to the original version. You should upload this as a separate file labeled 'Revised Manuscript with Track Changes'.
- An unmarked version of your revised paper without tracked changes. You should upload this as a separate file labeled 'Manuscript'.

If you would like to make changes to your financial disclosure, please include your updated statement in your cover letter. Guidelines for resubmitting your figure files are available below the reviewer comments at the end of this letter.

If applicable, we recommend that you deposit your laboratory protocols in protocols.io to enhance the reproducibility of your results. Protocols.io assigns your protocol its own identifier (DOI) so that it can be cited independently in the future. For instructions see: https://journals.plos.org/plosone/s/submission-guidelines#loc-laboratory-protocols. Additionally, PLOS ONE offers an option for publishing peer-reviewed Lab Protocol articles, which describe protocols hosted on protocols.io. Read more information on sharing protocols at https://plos.org/protocols?utm_medium=editorial-email&utm_source=authorletters&utm_campaign=protocols.

We look forward to receiving your revised manuscript.

Kind regards,

Abdelaziz Hendy, PHD

Academic Editor

PLOS ONE

Journal Requirements:

When submitting your revision, we need you to address these additional requirements.

1. Please ensure that your manuscript meets PLOS ONE's style requirements, including those for file naming. The PLOS ONE style templates can be found at

https://journals.plos.org/plosone/s/file?id=wjVg/PLOSOne_formatting_sample_main_body.pdf and

https://journals.plos.org/plosone/s/file?id=ba62/PLOSOne_formatting_sample_title_authors_affiliations.pdf

2. We note that the grant information you provided in the ‘Funding Information’ and ‘Financial Disclosure’ sections do not match.

When you resubmit, please ensure that you provide the correct grant numbers for the awards you received for your study in the ‘Funding Information’ section.

3. Thank you for stating the following financial disclosure:

“Iran University of Medical Sciences for the financial support (Number: 1401-2-25-23476)”

Please state what role the funders took in the study. If the funders had no role, please state: "The funders had no role in study design, data collection and analysis, decision to publish, or preparation of the manuscript."

If this statement is not correct you must amend it as needed.

Please include this amended Role of Funder statement in your cover letter; we will change the online submission form on your behalf.

4. Thank you for stating the following in the Acknowledgments Section of your manuscript:

“Authors would like to thank Iran University of Medical Sciences for the financial support (Number: 1401-2-25-23476). Also, thanks for the help of participants of this study. “

We note that you have provided funding information that is currently declared in your Funding Statement. However, funding information should not appear in the Acknowledgments section or other areas of your manuscript. We will only publish funding information present in the Funding Statement section of the online submission form.

Please remove any funding-related text from the manuscript and let us know how you would like to update your Funding Statement. Currently, your Funding Statement reads as follows:

“Iran University of Medical Sciences for the financial support (Number: 1401-2-25-23476)”

Please include your amended statements within your cover letter; we will change the online submission form on your behalf.

5. Please include captions for your Supporting Information files at the end of your manuscript, and update any in-text citations to match accordingly. Please see our Supporting Information guidelines for more information: http://journals.plos.org/plosone/s/supporting-information.

6. If the reviewer comments include a recommendation to cite specific previously published works, please review and evaluate these publications to determine whether they are relevant and should be cited. There is no requirement to cite these works unless the editor has indicated otherwise.

Additional Editor Comments:

You must perform concise grammar checks and editing, and apply all necessary reviewer modifications

Reviewer 1:

We sincerely thank the authors for their valuable efforts in preparing this manuscript, and we also extend our gratitude to the editor for the opportunity to review it. In order to help improve the overall quality of the work, we offer the following comments and suggestions.

Abstract

Comment 1: The abstract is generally well-structured but could benefit from greater specificity regarding the methodology (e.g., SEM phases like measurement vs. structural models).

Comment 2: Add explicit mention of theoretical contribution (e.g., "extends previous models of psychological capital…").

Comment 3: Please ensure that writing and spelling conventions are followed correctly in the abstract; for example, use "abstract" instead of "abstracta."

Introduction

Comment 4: The introduction offers a solid conceptual background, but the research gap should be more clearly articulated. Why is the moderating role of intolerance of uncertainty important in this context?

Comment 5: Consider reducing repetition in defining psychological capital and its components. Streamlining will enhance flow.

Comment 6: The theoretical framework and hypothesis development are currently interwoven within the Introduction. For clarity and better organization, it is recommended to present the theoretical framework and the development of hypotheses in two separate sub-sections. This will help readers follow the logical flow of concepts and understand how the research model and hypotheses are derived from existing literature.

Materials and Methods

Comment 7: Good detail on instruments. However, the rationale for selecting the sample size should be better justified beyond stating SEM needs (include power analysis if possible).

Comment 8: Clarify ethical procedures: you mention verbal consent in the text, but the abstract refers to written consent. This inconsistency needs resolution.

Comment 9: Detail on missing data handling or response rate is missing—please add.

Comment 10: Expand briefly on validity evidence of the questionnaires in the local (Iranian) context.

Results

Comment 11: Tables are useful, but figure captions should be more informative (e.g., "Structural Model Output using Smart-PLS").

Comment 12: The results narrative is descriptive but could benefit from more interpretive statements highlighting the implications of each statistical result.

Comment 13: Provide effect size interpretations alongside statistical significance where possible (e.g., is β = .378 considered strong?).

Comment 14: Consider restructuring Table 2 to improve readability—current formatting is difficult to interpret at a glance.

Discussion

Comment 15: The discussion effectively interprets results, but lacks critical engagement with conflicting studies or limitations of findings.

Comment 16: Theoretical contribution should be more explicitly stated—what does this add to literature beyond empirical confirmation?

Comment 17: Some paragraphs (e.g., on optimism) could be shortened or merged for better cohesion.

Comment 18: No policy or organizational recommendations are clearly formulated—these should be added given the applied nature of the study.

Conclusion

Comment 19: The conclusion is mostly a summary. It should more clearly reflect implications for hospital management, staff training, or policy.

Comment 20: No explicit limitations section is provided. Please add a paragraph discussing at least methodological limitations (e.g., cross-sectional design, self-report bias).

Comment 21: Suggestions for future research are absent. Include 1–2 directions (e.g., longitudinal studies, broader geographic samples).

Grammar and Writing Style

Comment 22: General English is clear but sometimes wordy or redundant (e.g., “refers to” used too frequently).

Comment 23: Inconsistent verb tenses—ensure uniform use of past tense in methods and results.

Comment 24: Some sentences are overly long (20+ words). Consider splitting for clarity and reader ease.

Comment 25: There are minor issues in article use and punctuation (e.g., “the psychological capital” vs. “psychological capital”).

Missing Sections

Comment 26: There is no separate limitations section—please add.

Comment 27: There are no clear policy implications or practical recommendations, despite this being an applied study. Add concrete suggestions (e.g., training modules, managerial practices).

Comment 28: No future research suggestions are included—these are essential for a scientific article.

Reviewer 2:

1. In the introduction section, accurate statistics on three main variables in the world and Iran should be mentioned.

2. In the introduction section, The study gap should be explained.

3. The introduction should mention why modeling was done and what is its importance in nursing?

4. The validity and reliability of the instruments inside and outside Iran, especially the construct validity, should be mentioned in the materials and methods section.

5. The sampling method should be explained in the methodology section.

6. It is essential to obtain written consent from participants and explain the purpose of the study to them.

7. Articles that are consistent with and inconsistent with the present study should be used in the discussion.

8. At the end of the discussion, limitations of the study and suggestions for nursing should be mentioned.

9. The references are fully updated and each them is written in full according to the journal format

1. In the introduction section, accurate statistics on three main variables in the world and Iran should be mentioned. 2. In the introduction section, The study gap should be explained. 3. The introduction should mention why modeling was done and what is its importance in nursing? 4. The validity and reliability of the instruments inside and outside Iran, especially the construct validity, should be mentioned in the materials and methods section. 5. The sampling method should be explained in the methodology section. 6. It is essential to obtain written consent from participants and explain the purpose of the study to them. 7. Articles that are consistent with and inconsistent with the present study should be used in the discussion. 8. At the end of the discussion, limitations of the study and suggestions for nursing should be mentioned. 9. The references are fully updated and each them is written in full according to the journal format

1. In the introduction section, accurate statistics on three main variables in the world and Iran should be mentioned.

2. In the introduction section, The study gap should be explained.

3. The introduction should mention why modeling was done and what is its importance in nursing?

4. The validity and reliability of the instruments inside and outside Iran, especially the construct validity, should be mentioned in the materials and methods section.

5. The sampling method should be explained in the methodology section.

6. It is essential to obtain written consent from participants and explain the purpose of the study to them.

7. Articles that are consistent with and inconsistent with the present study should be used in the discussion.

8. At the end of the discussion, limitations of the study and suggestions for nursing should be mentioned.

9. The references are fully updated and each them is written in full according to the journal format

[Note: HTML markup is below. Please do not edit.]

Reviewers' comments:

Reviewer's Responses to Questions

**Comments to the Author**

1. Is the manuscript technically sound, and do the data support the conclusions?

The manuscript must describe a technically sound piece of scientific research with data that supports the conclusions. Experiments must have been conducted rigorously, with appropriate controls, replication, and sample sizes. The conclusions must be drawn appropriately based on the data presented.

Reviewer #1: Partly

Reviewer #2: Partly

**********

2. Has the statistical analysis been performed appropriately and rigorously?

Reviewer #1: No

Reviewer #2: Yes

**********

3. Have the authors made all data underlying the findings in their manuscript fully available?

The PLOS Data policy requires authors to make all data underlying the findings described in their manuscript fully available without restriction, with rare exception (please refer to the Data Availability Statement in the manuscript PDF file). The data should be provided as part of the manuscript or its supporting information, or deposited to a public repository. For example, in addition to summary statistics, the data points behind means, medians and variance measures should be available. If there are restrictions on publicly sharing data—e.g. participant privacy or use of data from a third party—those must be specified.

Reviewer #1: No

Reviewer #2: Yes

**********

4. Is the manuscript presented in an intelligible fashion and written in standard English?

PLOS ONE does not copyedit accepted manuscripts, so the language in submitted articles must be clear, correct, and unambiguous. Any typographical or grammatical errors should be corrected at revision, so please note any specific errors here.

Reviewer #1: Yes

Reviewer #2: Yes

**********

5. Review Comments to the Author

Please use the space provided to explain your answers to the questions above. You may also include additional comments for the author, including concerns about dual publication, research ethics, or publication ethics. (Please upload your review as an attachment if it exceeds 20,000 characters)

Reviewer #1: We sincerely thank the authors for their valuable efforts in preparing this manuscript, and we also extend our gratitude to the editor for the opportunity to review it. In order to help improve the overall quality of the work, we offer the following comments and suggestions.

Abstract

Comment 1: The abstract is generally well-structured but could benefit from greater specificity regarding the methodology (e.g., SEM phases like measurement vs. structural models).

Comment 2: Add explicit mention of theoretical contribution (e.g., "extends previous models of psychological capital…").

Comment 3: Please ensure that writing and spelling conventions are followed correctly in the abstract; for example, use "abstract" instead of "abstracta."

Introduction

Comment 4: The introduction offers a solid conceptual background, but the research gap should be more clearly articulated. Why is the moderating role of intolerance of uncertainty important in this context?

Comment 5: Consider reducing repetition in defining psychological capital and its components. Streamlining will enhance flow.

Comment 6: The theoretical framework and hypothesis development are currently interwoven within the Introduction. For clarity and better organization, it is recommended to present the theoretical framework and the development of hypotheses in two separate sub-sections. This will help readers follow the logical flow of concepts and understand how the research model and hypotheses are derived from existing literature.

Materials and Methods

Comment 7: Good detail on instruments. However, the rationale for selecting the sample size should be better justified beyond stating SEM needs (include power analysis if possible).

Comment 8: Clarify ethical procedures: you mention verbal consent in the text, but the abstract refers to written consent. This inconsistency needs resolution.

Comment 9: Detail on missing data handling or response rate is missing—please add.

Comment 10: Expand briefly on validity evidence of the questionnaires in the local (Iranian) context.

Results

Comment 11: Tables are useful, but figure captions should be more informative (e.g., "Structural Model Output using Smart-PLS").

Comment 12: The results narrative is descriptive but could benefit from more interpretive statements highlighting the implications of each statistical result.

Comment 13: Provide effect size interpretations alongside statistical significance where possible (e.g., is β = .378 considered strong?).

Comment 14: Consider restructuring Table 2 to improve readability—current formatting is difficult to interpret at a glance.

Discussion

Comment 15: The discussion effectively interprets results, but lacks critical engagement with conflicting studies or limitations of findings.

Comment 16: Theoretical contribution should be more explicitly stated—what does this add to literature beyond empirical confirmation?

Comment 17: Some paragraphs (e.g., on optimism) could be shortened or merged for better cohesion.

Comment 18: No policy or organizational recommendations are clearly formulated—these should be added given the applied nature of the study.

Conclusion

Comment 19: The conclusion is mostly a summary. It should more clearly reflect implications for hospital management, staff training, or policy.

Comment 20: No explicit limitations section is provided. Please add a paragraph discussing at least methodological limitations (e.g., cross-sectional design, self-report bias).

Comment 21: Suggestions for future research are absent. Include 1–2 directions (e.g., longitudinal studies, broader geographic samples).

Grammar and Writing Style

Comment 22: General English is clear but sometimes wordy or redundant (e.g., “refers to” used too frequently).

Comment 23: Inconsistent verb tenses—ensure uniform use of past tense in methods and results.

Comment 24: Some sentences are overly long (20+ words). Consider splitting for clarity and reader ease.

Comment 25: There are minor issues in article use and punctuation (e.g., “the psychological capital” vs. “psychological capital”).

Missing Sections

Comment 26: There is no separate limitations section—please add.

Comment 27: There are no clear policy implications or practical recommendations, despite this being an applied study. Add concrete suggestions (e.g., training modules, managerial practices).

Comment 28: No future research suggestions are included—these are essential for a scientific article.

Reviewer #2: 1. In the introduction section, accurate statistics on three main variables in the world and Iran should be mentioned.

2. In the introduction section, The study gap should be explained.

3. The introduction should mention why modeling was done and what is its importance in nursing?

4. The validity and reliability of the instruments inside and outside Iran, especially the construct validity, should be mentioned in the materials and methods section.

5. The sampling method should be explained in the methodology section.

6. It is essential to obtain written consent from participants and explain the purpose of the study to them.

7. Articles that are consistent with and inconsistent with the present study should be used in the discussion.

8. At the end of the discussion, limitations of the study and suggestions for nursing should be mentioned.

9. The references are fully updated and each them is written in full according to the journal format

**********

6. PLOS authors have the option to publish the peer review history of their article (what does this mean?). If published, this will include your full peer review and any attached files.

If you choose “no”, your identity will remain anonymous but your review may still be made public.

**Do you want your identity to be public for this peer review?**   For information about this choice, including consent withdrawal, please see our Privacy Policy.

Reviewer #1: No

Reviewer #2: **Yes:** 1. In the introduction section, accurate statistics on three main variables in the world and Iran should be mentioned.

2. In the introduction section, The study gap should be explained.

3. The introduction should mention why modeling was done and what is its importance in nursing?

4. The validity and reliability of the instruments inside and outside Iran, especially the construct validity, should be mentioned in the materials and methods section.

5. The sampling method should be explained in the methodology section.

6. It is essential to obtain written consent from participants and explain the purpose of the study to them.

7. Articles that are consistent with and inconsistent with the present study should be used in the discussion.

8. At the end of the discussion, limitations of the study and suggestions for nursing should be mentioned.

9. The references are fully updated and each them is written in full according to the journal format

**********

[NOTE: If reviewer comments were submitted as an attachment file, they will be attached to this email and accessible via the submission site. Please log into your account, locate the manuscript record, and check for the action link "View Attachments". If this link does not appear, there are no attachment files.]

While revising your submission, please upload your figure files to the Preflight Analysis and Conversion Engine (PACE) digital diagnostic tool, https://pacev2.apexcovantage.com/. PACE helps ensure that figures meet PLOS requirements. To use PACE, you must first register as a user. Registration is free. Then, login and navigate to the UPLOAD tab, where you will find detailed instructions on how to use the tool. If you encounter any issues or have any questions when using PACE, please email PLOS at figures@plos.org. Please note that Supporting Information files do not need this step.

# Author comment: Author response to Decision Letter 1

**DOI:** 10.1371/journal.pone.0350761.r002

27 Aug 2025

Dear Editor. Prof. Abdelaziz Hendy

We would like to express our sincere gratitude to the Editor for the time and effort dedicated to handling our manuscript. We have carefully considered all the reviewers’ comments and the journal’s requirements, and we have thoroughly revised the manuscript accordingly. We believe that these revisions have significantly improved the clarity, rigor, and overall quality of the paper, and we hope that the revised version will now be suitable for publication in PLOS ONE.

General Response to Reviewers

We would like to sincerely thank both reviewers for their valuable and constructive comments. Their feedback greatly helped us to improve the clarity, scientific rigor, and overall quality of our manuscript. To facilitate readability, we highlighted all revisions in the manuscript using a single color, rather than separate colors for each reviewer, because several of the comments overlapped or addressed similar issues. This approach ensured a more coherent presentation of the changes.

In particular, since multiple comments from both reviewers related to the Introduction (e.g., providing clearer justification, adding relevant references, addressing conflicting evidence, and improving conciseness), we found it necessary to substantially rewrite the entire Introduction section. This allowed us to integrate the reviewers’ suggestions more effectively and to present a logically structured and updated rationale for the study.

We have also carefully addressed all other specific comments throughout the manuscript. For each point raised, we provide a detailed, point-by-point response below, indicating the corresponding changes made in the revised version.

Reviewer 1:

We sincerely thank the authors for their valuable efforts in preparing this manuscript, and we also extend our gratitude to the editor for the opportunity to review it. In order to help improve the overall quality of the work, we offer the following comments and suggestions.

Abstract

Comment 1: The abstract is generally well-structured but could benefit from greater specificity regarding the methodology (e.g., SEM phases like measurement vs. structural models).

Comment 2: Add explicit mention of theoretical contribution (e.g., "extends previous models of psychological capital…").

Comment 3: Please ensure that writing and spelling conventions are followed correctly in the abstract; for example, use "abstract" instead of "abstracta."

We sincerely thank the reviewer for the constructive feedback regarding the abstract and keywords. In this revision, the abstract has been carefully rewritten to provide greater methodological specificity by explicitly distinguishing between the measurement and structural phases of SEM. We also added a clear statement on the theoretical contribution, highlighting that this study extends previous models of psychological capital by incorporating intolerance of uncertainty as a moderator. Minor spelling and formatting issues (e.g., “Abstracta” corrected to “Abstract”) were also addressed. Furthermore, the keywords have been revised in accordance with MeSH terminology: “Psychological Capital” was aligned with Psychology, Positive, “Job Performance” corrected to Work Performance, and “Intolerance of Uncertainty” revised to Uncertainty, alongside Nurses and Self-Efficacy. These revisions ensure both improved clarity and compliance with indexing standards.

Introduction

Comment 4: The introduction offers a solid conceptual background, but the research gap should be more clearly articulated. Why is the moderating role of intolerance of uncertainty important in this context?

We thank the reviewer for this insightful comment. The Introduction has been revised to more clearly articulate the research gap. Specifically, we highlighted that although psychological capital (PsyCap) is known to improve job performance, healthcare environments are often characterized by ambiguity and unpredictability, making intolerance of uncertainty (IU) an important contextual factor. We now explain how IU may weaken the benefits of PsyCap, thereby clarifying why the moderating role of IU is critical to examine.

Comment 5: Consider reducing repetition in defining psychological capital and its components. Streamlining will enhance flow.

We agree with the reviewer. The definition of psychological capital (PsyCap) and its components (self-efficacy, optimism, hope, resilience) has been streamlined to avoid unnecessary repetition. This revision improves the flow of the text and ensures greater clarity.

Comment 6: The theoretical framework and hypothesis development are currently interwoven within the Introduction. For clarity and better organization, it is recommended to present the theoretical framework and the development of hypotheses in two separate sub-sections. This will help readers follow the logical flow of concepts and understand how the research model and hypotheses are derived from existing literature.

Thank you for this helpful suggestion. Following the reviewer’s recommendation, we have reorganized the Introduction by creating two distinct subsections: “Theoretical Framework” and “Hypothesis Development.” This separation enhances readability and provides a clearer logical flow for readers.

Materials and Methods

Comment 7: Good detail on instruments. However, the rationale for selecting the sample size should be better justified beyond stating SEM needs (include power analysis if possible).

We sincerely appreciate the reviewer’s valuable comment regarding the rationale for sample size. In the revised manuscript, we have expanded this section to provide a stronger justification. Specifically, we explained that according to Kline (2016), approximately 10 participants are required per estimated parameter. Given our model with 18 free parameters, the minimum sample size should be around 180. In addition, Hair et al. (2019) recommend a minimum of 200 participants for complex SEM models. Our final sample of 212 nurses exceeds all of these thresholds, ensuring sufficient statistical power and robustness of the SEM results.

Comment 8: Clarify ethical procedures: you mention verbal consent in the text, but the abstract refers to written consent. This inconsistency needs resolution.

Thank you for pointing out the inconsistency. This was a typographical error in the abstract. The study obtained verbal informed consent, not written consent. The abstract has been corrected to state “verbal informed consent,” consistent with the Methods section. We also clarified that verbal consent was obtained in accordance with the ethics approval granted by the Iran University of Medical Sciences (IR.IUMS.REC.1401.561).

Comment 9: Detail on missing data handling or response rate is missing—please add.

Thank you for the helpful comment. We have added a dedicated subsection, Response Rate and Missing Data, to the Methods. It reports that 235 nurses were approached and 212 completed the survey (90.2% response rate). All returned questionnaires were complete after on-site checks; therefore, no item-level missing data were present and no imputation procedures were required.

Comment 10: Expand briefly on validity evidence of the questionnaires in the local (Iranian) context.

We appreciate this helpful request. We have now added a concise paragraph in the Methods summarizing Iranian psychometric evidence for all instruments. Specifically, we cite a Persian validation of PCQ-24 reporting α=0.85–0.89 and test–retest r=0.80 , a Persian validation of IUS-12 confirming a two-factor structure with CR=0.86 and α=0.89 , and Iranian studies using the Paterson Job Performance Questionnaire with α≈0.85–0.90 and split-half≈0.85. These additions clarify that the questionnaires have acceptable validity and reliability in the local context.

Results

Comment 11: Tables are useful, but figure captions should be more informative (e.g., "Structural Model Output using Smart-PLS").

We appreciate the reviewer’s suggestion. We have revised the figure captions to be more informative. The updated captions now specify the software (SmartPLS 3), clarify whether the figure depicts the conceptual or structural model, report standardized path coefficients and their significance (from 5,000-sample bootstrapping), indicate the sample size (N=212), and define all abbreviations. Measurement-model details are referenced to Table 3 to avoid clutter.

Comment 12: The results narrative is descriptive but could benefit from more interpretive statements highlighting the implications of each statistical result.

Thank you for this helpful suggestion. We have revised the Results section to add concise interpretive statements after each statistical finding. In particular, we (i) comment on the magnitude and practical meaning of key coefficients (e.g., the moderate PsyCap→performance effect and the negative moderation by intolerance of uncertainty), (ii) explain what reliability/validity indices imply for construct quality, and (iii) clarify the predictive relevance of the model (Q²). These additions guide readers from numeric results to their substantive implications for nursing practice and future interventions.

Comment 13: Provide effect size interpretations alongside statistical significance where possible (e.g., is β = .378 considered strong?).

Thank you for the suggestion. We have added effect-size interpretations alongside p-values. Following Hair et al., standardized path coefficients of ≈0.10, 0.30, and 0.50 indicate small, medium, and large effects; thus, the PsyCap→performance path (β = 0.378) reflects a medium effect, and the moderation by IU (β = −0.161) is small-to-medium. We also clarify the practical implications of these effects in the Results narrative.

Comment 14: Consider restructuring Table 2 to improve readability—current formatting is difficult to interpret at a glance.

Thank you for this helpful suggestion. We have restructured Table 2 into a compact correlation matrix that presents Pearson’s r and the corresponding two-tailed p-values in each cell, with clear row/column headers, defined abbreviations, and the sample size. Redundant “Significance level” rows were removed to improve glance readability. Significant correlations are highlighted with symbols and explained in the table note.

Discussion

Comment 15: The discussion effectively interprets results, but lacks critical engagement with conflicting studies or limitations of findings.

Thank you for this important observation. We revised the Discussion to (i) add a dedicated paragraph that critically contrasts our findings with studies reporting weaker or non-significant PsyCap–performance effects and alternative patterns for intolerance of uncertainty (IU), and (ii) expand the limitations to address design and measurement constraints. We now discuss potential reasons for discrepancies across studies (e.g., performance operationalization, unit context and demands, leadership climate, and method differences), and we outline concrete implications for interpretation and future research. These additions are included under the new subheadings “Conflicting evidence and alternative explanations” and “Limitations and future research.”

Comment 16: Theoretical contribution should be more explicitly stated—what does this add to literature beyond empirical confirmation?

Thank you for this valuable point. We have now made the theoretical contribution explicit in a dedicated paragraph. Beyond empirical confirmation, our study (i) identifies intolerance of uncertainty (IU) as a boundary condition that attenuates the positive PsyCap→performance link, (ii) integrates positive organizational behavior accounts of PsyCap (e.g., efficacy/hope-driven agency) with stress-and-coping/uncertainty perspectives (threat appraisal → avoidance/over-checking), and (iii) offers explanatory leverage for mixed findings in prior work by specifying when PsyCap’s resources translate—or fail to translate—into performance. This yields testable predictions (dual-path interventions that both build PsyCap and reduce IU) and a context-sensitive framework for nursing performance. The paragraph has been added under the subheading “Theoretical contribution.”

Comment 17: Some paragraphs (e.g., on optimism) could be shortened or merged for better cohesion.

Thank you for the helpful suggestion. We fully agree that cohesion is important. After carefully revisiting the Discussion, we retained the original paragraph structure because it mirrors the logic of our model (PsyCap components → structural findings → moderation by IU → implications) and preserves clear traceability from results to theory. Instead of a major re-organization, we streamlined the prose by removing redundancies, tightened the optimism paragraph, and added brief transition sentences to improve flow. We believe the revised text enhances readability while maintaining the necessary conceptual distinctions that would be blurred by merging larger sections.

Comment 18: No policy or organizational recommendations are clearly formulated—these should be added given the applied nature of the study.

Thank you for this valuable suggestion. We have added a dedicated subsection entitled “Practical Implications and Policy/Organizational Recommendations” to translate our findings into actionable steps. The recommendations are structured across policy, organizational (hospital), and unit/manager levels, specify responsible stakeholders, outline mechanisms of action (building PsyCap; reducing uncertainty/IU), and propose monitoring indicators for implementation. We believe these additions address the applied nature of the study and provide clear guidance for decision-makers.

Conclusion

Comment 19: The conclusion is mostly a summary. It should more clearly reflect implications for hospital management, staff training, or policy.

We thank the reviewer for this insightful comment. We have revised the Conclusion section to move beyond summarizing results and to highlight actionable implications for hospital management, staff training, and policy. Specifically, the revised conclusion now (i) emphasizes the role of hospital managers in implementing PsyCap-development programs, (ii) underlines the importance of embedding uncertainty-management strategies into staff training, and (iii) proposes policy-level recommendations for integrating psychological resources into accreditation and workforce standards. These revisions are located in the Conclusion section

Comment 20: No explicit limitations section is provided. Please add a paragraph discussing at least methodological limitations (e.g., cross-sectional design, self-report bias).

We agree with the reviewer’s observation. In response, we have added a dedicated Limitations paragraph at the end of the manuscript. This new section acknowledges the methodological limitations, including the cross-sectional design, potential self-report bias, restricted generalizability, and the absence of contextual variables such as leadership or workload.

Comment 21: Suggestions for future research are absent. Include 1–2 directions (e.g., longitudinal studies, broader geographic samples).

We thank the reviewer for this helpful suggestion. In response, we have expanded the Limitations section to include explicit recommendations for future research. Specifically, we now propose longitudinal and interventional studies to strengthen causal inference, as well as the inclusion of broader geographic and organizational samples to enhance generalizability. We also suggest using multi-source performance data to complement self-reports. These additions can be found in the revised Limitations and Future Research section

Grammar and Writing Style

Comment 22: General English is clear but sometimes wordy or redundant (e.g., “refers to” used too frequently).

We appreciate the reviewer’s observation. In response, we have carefully revised the manuscript to reduce redundancy and improve conciseness. Specifically, we replaced repetitive expressions such as “refers to” with more varied wording (e.g., “denotes,” “is defined as,” “represents”), simplified lengthy sentences, and eliminated unnecessary transitional phrases. These edits enhance readability while preserving the accuracy of the scientific content.

Comment 23: Inconsi

Attachment Submitted filename: Our reply to reviewers.docx

# Peer review: Decision Letter 1

**DOI:** 10.1371/journal.pone.0350761.r003

**Authors:** Jordan Llego

19 Dec 2025

PONE-D-25-35558R1

Modeling the Effect of Psychological Capital on Nurses' Job Performance with the Moderating Role of Intolerance of Uncertainty

PLOS One

Dear Dr. Ebrahimi,

Thank you for submitting your manuscript to PLOS ONE. After careful consideration, we feel that it has merit but does not fully meet PLOS ONE’s publication criteria as it currently stands. Therefore, we invite you to submit a revised version of the manuscript that addresses the points raised during the review process.

Please submit your revised manuscript by Feb 02 2026 11:59PM. If you will need more time than this to complete your revisions, please reply to this message or contact the journal office at plosone@plos.org. When you're ready to submit your revision, log on to https://www.editorialmanager.com/pone/ and select the 'Submissions Needing Revision' folder to locate your manuscript file.

Please include the following items when submitting your revised manuscript:

- A letter that responds to each point raised by the academic editor and reviewer(s). You should upload this letter as a separate file labeled 'Response to Reviewers'.
- A marked-up copy of your manuscript that highlights changes made to the original version. You should upload this as a separate file labeled 'Revised Manuscript with Track Changes'.
- An unmarked version of your revised paper without tracked changes. You should upload this as a separate file labeled 'Manuscript'.

If you would like to make changes to your financial disclosure, please include your updated statement in your cover letter. Guidelines for resubmitting your figure files are available below the reviewer comments at the end of this letter.

If applicable, we recommend that you deposit your laboratory protocols in protocols.io to enhance the reproducibility of your results. Protocols.io assigns your protocol its own identifier (DOI) so that it can be cited independently in the future. For instructions see: https://journals.plos.org/plosone/s/submission-guidelines#loc-laboratory-protocols. Additionally, PLOS ONE offers an option for publishing peer-reviewed Lab Protocol articles, which describe protocols hosted on protocols.io. Read more information on sharing protocols at https://plos.org/protocols?utm_medium=editorial-email&utm_source=authorletters&utm_campaign=protocols.

We look forward to receiving your revised manuscript.

Kind regards,

Jordan Llego, PhD ELM, D. Hon. Ex., PhDN, RN

Academic Editor

PLOS One

Journal Requirements:

1. If the reviewer comments include a recommendation to cite specific previously published works, please review and evaluate these publications to determine whether they are relevant and should be cited. There is no requirement to cite these works unless the editor has indicated otherwise.

Additional Editor Comments:

Alongside the reviewer’s helpful feedback, there are a few additional editorial points to consider to meet PLOS ONE’s standards. Your use of structural equation modeling is appropriate, but the Methods section would be even clearer if you stated up front whether your SEM approach was strictly confirmatory or partly exploratory, especially since you included a moderating path. Clarifying this will help readers understand the scope of your findings and avoid any confusion about model fit and theoretical confirmation.

Please also double-check the ethics documentation for consistency. While your manuscript now correctly notes that verbal informed consent was obtained, the submission system still mentions written consent. Make sure this is updated everywhere—including in your submission metadata—to avoid any last-minute ethical compliance concerns. Also, confirm that your ethics committee’s approval explicitly covers the waiver of written consent, and that this is reflected throughout your materials.

For transparency, it would be helpful to briefly mention how you addressed or considered common method bias, since all your data came from self-report instruments at one time point. Even if you didn’t run a formal statistical test, a short acknowledgment—such as describing steps like ensuring anonymity or separating scales—will strengthen both your Methods and Limitations sections.

In the Results and Discussion, your explanations of effect sizes and moderation are clear. However, some passages use language that could be read as causal or prescriptive. Since PLOS ONE values methodological neutrality, please make sure all interpretations are framed as associative and conditional relationships—not causal—especially when talking about interventions or policy implications.

Finally, your Discussion section is thorough, but its length may make it harder for readers to see your main message. I suggest sharpening the focus by clearly restating how your study moves the field forward—especially by showing how intolerance of uncertainty acts as a boundary condition in PsyCap–performance research. Adding a concise summary paragraph near the end of the Discussion will help highlight your study’s unique value.

[Note: HTML markup is below. Please do not edit.]

Reviewer's Responses to Questions

**Comments to the Author**

1. If the authors have adequately addressed your comments raised in a previous round of review and you feel that this manuscript is now acceptable for publication, you may indicate that here to bypass the “Comments to the Author” section, enter your conflict of interest statement in the “Confidential to Editor” section, and submit your "Accept" recommendation.

Reviewer #1: (No Response)

**********

2. Is the manuscript technically sound, and do the data support the conclusions?

The manuscript must describe a technically sound piece of scientific research with data that supports the conclusions. Experiments must have been conducted rigorously, with appropriate controls, replication, and sample sizes. The conclusions must be drawn appropriately based on the data presented.

Reviewer #1: (No Response)

**********

3. Has the statistical analysis been performed appropriately and rigorously?

Reviewer #1: (No Response)

**********

4. Have the authors made all data underlying the findings in their manuscript fully available?

The PLOS Data policy requires authors to make all data underlying the findings described in their manuscript fully available without restriction, with rare exception (please refer to the Data Availability Statement in the manuscript PDF file). The data should be provided as part of the manuscript or its supporting information, or deposited to a public repository. For example, in addition to summary statistics, the data points behind means, medians and variance measures should be available. If there are restrictions on publicly sharing data—e.g. participant privacy or use of data from a third party—those must be specified.

Reviewer #1: (No Response)

**********

5. Is the manuscript presented in an intelligible fashion and written in standard English?

PLOS ONE does not copyedit accepted manuscripts, so the language in submitted articles must be clear, correct, and unambiguous. Any typographical or grammatical errors should be corrected at revision, so please note any specific errors here.

Reviewer #1: (No Response)

**********

6. Review Comments to the Author

Please use the space provided to explain your answers to the questions above. You may also include additional comments for the author, including concerns about dual publication, research ethics, or publication ethics. (Please upload your review as an attachment if it exceeds 20,000 characters)

Reviewer #1: Thank you very much, Professor, for the opportunity to review this manuscript.

My comments for revision are as follows:

General Comments

The manuscript is well-structured and scientifically solid; however, some sections need tightening for clarity, consistency, and stronger alignment with PLOS ONE’s reporting standards.

Please ensure consistent terminology for constructs (e.g., “psychological capital” vs. “PsyCap”) and for consent type (“verbal” vs. “written”) across all sections.

Abstract

1. The first sentence could explicitly state the design (e.g., “a cross-sectional analytical study”) for methodological clarity.

2. Consider shortening the last two sentences to emphasize implications more succinctly.

3. Ensure that all abbreviations (e.g., SEM, IU) are defined at first mention.

Introduction

1. The introduction is comprehensive, but the flow can be improved by reducing background repetition on PsyCap.

2. The research gap could be highlighted earlier (around line 80–85) to strengthen reader engagement.

3. Figure 1 should be referenced with a more explicit description in text (e.g., “Figure 1 illustrates the proposed moderating framework…”).

Methods

1. The section is detailed and clear. However, the ethics paragraph still inconsistently refers to written consent in the ethics statement while “verbal consent” is noted in the main text. This should be reconciled to match ethics committee approval.

2. The sampling procedure description is excellent, but please move some technical detail (SPSS commands, oversampling explanation) to a supplementary appendix for brevity.

3. Clarify whether any control variables (e.g., gender, age, experience) were tested in the SEM model.

4. Add brief justification for using the 27-item IUS rather than the IUS-12 short form, since the latter is more common in nursing research.

Results

1. Figures and tables are informative but need uniform style (same decimal precision and table notes).

2. Provide sample size (N=212) in every table caption.

3. The interpretation sentences following Tables 2 and 4 could more clearly relate numeric values to the study hypotheses (e.g., “Thus, H1 was supported, while H2 was not…”).

4. Report R² and f² values for main paths to support the discussion on effect size and model strength.

Discussion

1. The discussion is strong but should better distinguish between empirical findings and interpretive commentary—currently they blend in several paragraphs.

2. When citing conflicting evidence, specify study contexts (e.g., countries, healthcare settings) to enrich the comparative analysis.

3. Add a short paragraph linking findings to existing nursing performance theories or models (e.g., Job Demands–Resources framework).

4. The “Practical Implications” subsection is excellent but could be condensed to highlight 3–4 priority recommendations.

Conclusion

1. Rephrase to avoid restating numerical findings; focus on implications for practice, management, and policy.

2. The final sentence could more directly state the contribution: “This study provides an empirically validated model explaining how intolerance of uncertainty moderates the effect of psychological capital on nurses’ performance.”

Limitations and Future Research

1. Well-written, but should add a note about the cultural context (Iranian hospitals) as a possible boundary to generalization.

2. Suggest specifying how longitudinal or intervention designs might operationalize PsyCap or IU changes.

References

1. Check consistency of et al. usage (journal requires up to 6 authors before “et al.”).

2. Ensure uniform citation style (e.g., spacing before volume and issue numbers).

3. Verify the accuracy of Iranian journal references (some may lack DOIs).

Language and Style

1. The manuscript is generally fluent but occasionally verbose; shorten sentences over 25 words.

2. Ensure consistent tense use—Methods and Results in past tense; Introduction and Discussion in present tense where applicable.

3. Avoid redundancy in transitions (e.g., remove phrases like “it should be noted that…”).

Best Regards

**********

7. PLOS authors have the option to publish the peer review history of their article (what does this mean?). If published, this will include your full peer review and any attached files.

If you choose “no”, your identity will remain anonymous but your review may still be made public.

**Do you want your identity to be public for this peer review?**   For information about this choice, including consent withdrawal, please see our Privacy Policy.

Reviewer #1: No

**********

[NOTE: If reviewer comments were submitted as an attachment file, they will be attached to this email and accessible via the submission site. Please log into your account, locate the manuscript record, and check for the action link "View Attachments". If this link does not appear, there are no attachment files.]

To ensure your figures meet our technical requirements, please review our figure guidelines: https://journals.plos.org/plosone/s/figures

You may also use PLOS’s free figure tool, NAAS, to help you prepare publication quality figures: https://journals.plos.org/plosone/s/figures#loc-tools-for-figure-preparation.

NAAS will assess whether your figures meet our technical requirements by comparing each figure against our figure specifications.

# Author comment: Author response to Decision Letter 2

**DOI:** 10.1371/journal.pone.0350761.r004

7 Jan 2026

Dear Editor

We would like to express our sincere gratitude to the Editor for the time and effort dedicated to handling our manuscript. We have carefully considered all the reviewers’ comments and the journal’s requirements, and we have thoroughly revised the manuscript accordingly. We believe that these revisions have significantly improved the clarity, rigor, and overall quality of the paper, and we hope that the revised version will now be suitable for publication in PLOS ONE.

Response to Reviewers

Reviewer 1:

The manuscript is well-structured and scientifically solid; however, some sections need tightening for clarity, consistency, and stronger alignment with PLOS ONE’s reporting standards.

Please ensure consistent terminology for constructs (e.g., “psychological capital” vs. “PsyCap”) and for consent type (“verbal” vs. “written”) across all sections.

Response: We thank the reviewer for the positive evaluation of the manuscript and for this helpful comment. In response, we carefully revised the manuscript to improve clarity and consistency in terminology in line with PLOS ONE reporting standards. Specifically, we standardized the use of terminology for key constructs by consistently using psychological capital (PsyCap) at first mention and PsyCap thereafter throughout the manuscript. In addition, the type of informed consent has been consistently reported as verbal informed consent across all relevant sections (Abstract, Methods, and Ethics statement). These revisions have enhanced clarity and ensured consistency throughout the manuscript.

Abstract

1. The first sentence could explicitly state the design (e.g., “a cross-sectional analytical study”) for methodological clarity.

Response: The first sentence of the Abstract was revised to explicitly state the study design as a cross-sectional analytical study to improve methodological clarity.

2. Consider shortening the last two sentences to emphasize implications more succinctly.

Response: The final two sentences of the Abstract were condensed to more succinctly emphasize the key practical implications of the findings.

3. Ensure that all abbreviations (e.g., SEM, IU) are defined at first mention.

Response: All abbreviations, including intolerance of uncertainty (IU) and structural equation modeling (SEM), are now defined at first mention in the Abstract.

Introduction

1. The introduction is comprehensive, but the flow can be improved by reducing background repetition on PsyCap.

Response: We thank the reviewer for this valuable suggestion. To improve the flow of the Introduction, we reduced repetitive background information on psychological capital by consolidating its definition and empirical evidence into a single, concise paragraph. This revision preserves the theoretical foundation while improving readability and narrative coherence.

2. The research gap could be highlighted earlier (around line 80–85) to strengthen reader engagement.

Response: In response to this comment, we revised the Introduction to explicitly highlight the research gap at an earlier stage. Specifically, we emphasized the lack of studies examining intolerance of uncertainty as a moderating condition in the relationship between psychological capital and nurses’ job performance, thereby strengthening the rationale and engagement for the present study.

3. Figure 1 should be referenced with a more explicit description in text (e.g., “Figure 1 illustrates the proposed moderating framework…”).

Response: We have revised the manuscript to include a clearer and more explicit in-text reference to Figure 1. The text now explicitly states that Figure 1 illustrates the proposed conceptual and moderating framework linking psychological capital, intolerance of uncertainty, and nurses’ job performance.

Methods

1. The section is detailed and clear. However, the ethics paragraph still inconsistently refers to written consent in the ethics statement while “verbal consent” is noted in the main text. This should be reconciled to match ethics committee approval.

Response: We thank the reviewer for pointing out this inconsistency. We carefully revised the ethics paragraph to ensure full consistency across the manuscript. All references to informed consent now clearly and consistently state that verbal informed consent was obtained from all participants, in accordance with the approval of the ethics committee.

2. The sampling procedure description is excellent, but please move some technical detail (SPSS commands, oversampling explanation) to a supplementary appendix for brevity.

Response: We thank the reviewer for the positive evaluation of the sampling procedure. In response to this comment, we streamlined the sampling description in the Methods section for brevity and clarity, while moving technical details (e.g., SPSS commands and oversampling procedures) to a supplementary appendix.

3. Clarify whether any control variables (e.g., gender, age, experience) were tested in the SEM model.

Response: We clarified this point in the Methods section. As the primary aim of the study was theory-driven hypothesis testing, the structural model was specified to focus on the hypothesized relationships among the core constructs. To preserve theoretical clarity and model parsimony, demographic variables were not included as control variables in the SEM.

4. Add brief justification for using the 27-item IUS rather than the IUS-12 short form, since the latter is more common in nursing research.

Response: We added a brief justification in the Methods section for using the 27-item version of the Intolerance of Uncertainty Scale. The IUS-27 was selected to provide more comprehensive construct coverage and due to its strong psychometric validation in Persian samples, which was particularly important for the moderation analysis.

Results

1. Figures and tables are informative but need uniform style (same decimal precision and table notes).

Response: We thank the reviewer for this helpful comment. All tables were carefully revised to ensure a uniform presentation style throughout the Results section. Specifically, decimal precision was standardized across all numerical values, p-values were reported consistently (e.g., p < 0.001), and table notes were harmonized using a single, consistent format in line with PLOS ONE reporting standards.

2. Provide sample size (N=212) in every table caption.

Response: In response to this comment, the sample size (N = 212) has been added to the captions of all tables to improve clarity and transparency.

3. The interpretation sentences following Tables 2 and 4 could more clearly relate numeric values to the study hypotheses (e.g., “Thus, H1 was supported, while H2 was not…”).

Response: We revised the interpretation sentences following Tables 2 and 4 to explicitly link the reported statistical results to the study hypotheses. The Results section now clearly states which hypotheses were supported and which were not, based on the numerical findings.

4. Report R² and f² values for main paths to support the discussion on effect size and model strength.

Response: In response to this comment, we added R² and f² values to the Results section to provide additional information on model explanatory power and effect sizes of the main and interaction paths. These additions strengthen the interpretation of model robustness and effect magnitude.

Discussion

1. The discussion is strong but should better distinguish between empirical findings and interpretive commentary—currently they blend in several paragraphs.

Response: We thank the reviewer for this constructive comment. The Discussion section was substantially revised to clearly distinguish between empirical findings and interpretive commentary. Specifically, we separated the neutral summary of the main results from their theoretical interpretation and comparison with prior studies, thereby improving clarity, structure, and narrative flow.

2. When citing conflicting evidence, specify study contexts (e.g., countries, healthcare settings) to enrich the comparative analysis.

Response: In response to this comment, we revised the Discussion to explicitly specify study contexts when discussing conflicting evidence. The revised text now highlights differences in countries, healthcare settings, and clinical environments to provide a more nuanced and context-sensitive comparative analysis.

3. Add a short paragraph linking findings to existing nursing performance theories or models (e.g., Job Demands–Resources framework).

Response: We added a new paragraph linking the study findings to established nursing performance theories, specifically the Job Demands–Resources (JD–R) framework. This addition situates psychological capital and intolerance of uncertainty within a theory-driven model of job performance, strengthening the conceptual contribution of the study.

4. The “Practical Implications” subsection is excellent but could be condensed to highlight 3–4 priority recommendations.

Response: The Practical Implications subsection was revised and condensed to highlight four priority recommendations. These focused recommendations emphasize psychological capital development, uncertainty management, supportive leadership practices, and organizational strategies to enhance nurses’ job performance.

Conclusion

1. Rephrase to avoid restating numerical findings; focus on implications for practice, management, and policy.

Response: We thank the reviewer for this helpful suggestion. The Conclusion section was revised to avoid restating numerical or statistical findings and to place greater emphasis on implications for clinical practice, management, and policy. The revised text now highlights the broader relevance of the findings for nursing performance improvement and healthcare system development.

2. The final sentence could more directly state the contribution: “This study provides an empirically validated model explaining how intolerance of uncertainty moderates the effect of psychological capital on nurses’ performance.”

Response: We revised the final sentence of the Conclusion to more explicitly state the study’s contribution. The Conclusion now clearly emphasizes that this study provides an empirically validated model explaining how intolerance of uncertainty moderates the effect of psychological capital on nurses’ job performance.

Limitations and Future Research

1. Well-written, but should add a note about the cultural context (Iranian hospitals) as a possible boundary to generalization.

Response: We thank the reviewer for this valuable suggestion. We revised the Limitations section to explicitly acknowledge the cultural and organizational context of Iranian hospitals as a potential boundary condition for generalizing the findings to other regions and healthcare systems.

2. Suggest specifying how longitudinal or intervention designs might operationalize PsyCap or IU changes.

Response: In response to this comment, we expanded the Future Research section to specify how longitudinal and intervention-based designs could operationalize changes in psychological capital and intolerance of uncertainty. The revised text now outlines potential PsyCap development and uncertainty-management interventions and highlights their value for clarifying causal pathways.

References

1. Check consistency of et al. usage (journal requires up to 6 authors before “et al.”).

Response: We revised the reference list to ensure consistent use of “et al.” in accordance with the journal’s guidelines, listing up to six authors before using “et al.” where applicable.

2. Ensure uniform citation style (e.g., spacing before volume and issue numbers).

Response: The reference list was carefully reviewed and edited to ensure a uniform citation style throughout, including consistent spacing, punctuation, and formatting of volume and issue numbers, in line with PLOS ONE requirements.

3. Verify the accuracy of Iranian journal references (some may lack DOIs).

Response: We verified the accuracy of all references, with particular attention to Iranian journal articles. For references without DOIs, complete and accurate bibliographic information has been provided in accordance with the journal’s citation guidelines.

Language and Style

1. The manuscript is generally fluent but occasionally verbose; shorten sentences over 25 words.

Response: We revised the manuscript to improve conciseness by shortening long sentences and reducing unnecessary wording across sections, while preserving meaning and scientific clarity.

2. Ensure consistent tense use—Methods and Results in past tense; Introduction and Discussion in present tense where applicable.

Response: We standardized verb tense throughout the manuscript. Methods and Results were edited to consistently use past tense, and Introduction and Discussion were revised to use present tense where appropriate, particularly for interpretation and general statements.

3. Avoid redundancy in transitions (e.g., remove phrases like “it should be noted that…”).

Response: We edited transitional phrases to eliminate redundancy and improve readability, removing unnecessary filler expressions and streamlining paragraph openings and linking sentences.

Attachment Submitted filename: Our_reply_to_reviewers_auresp_2.docx

# Editor report: Decision Letter 2

**DOI:** 10.1371/journal.pone.0350761.r005

**Authors:** Jordan Llego

25 Mar 2026

<div>PONE-D-25-35558R2

Modeling the Effect of Psychological Capital on Nurses' Job Performance with the Moderating Role of Intolerance of Uncertainty

PLOS One

Dear Dr. Ebrahimi,

Thank you for submitting your manuscript to PLOS ONE. After careful consideration, we feel that it has merit but does not fully meet PLOS ONE’s publication criteria as it currently stands. Therefore, we invite you to submit a revised version of the manuscript that addresses the points raised during the review process.

Please submit your revised manuscript by May 09 2026 11:59PM. If you will need more time than this to complete your revisions, please reply to this message or contact the journal office at plosone@plos.org. When you're ready to submit your revision, log on to https://www.editorialmanager.com/pone/ and select the 'Submissions Needing Revision' folder to locate your manuscript file.

Please include the following items when submitting your revised manuscript:

- A letter that responds to each point raised by the academic editor and reviewer(s). You should upload this letter as a separate file labeled 'Response to Reviewers'.
- A marked-up copy of your manuscript that highlights changes made to the original version. You should upload this as a separate file labeled 'Revised Manuscript with Track Changes'.
- An unmarked version of your revised paper without tracked changes. You should upload this as a separate file labeled 'Manuscript'.

If you would like to make changes to your financial disclosure, please include your updated statement in your cover letter. Guidelines for resubmitting your figure files are available below the reviewer comments at the end of this letter.

If applicable, we recommend that you deposit your laboratory protocols in protocols.io to enhance the reproducibility of your results. Protocols.io assigns your protocol its own identifier (DOI) so that it can be cited independently in the future. For instructions see: https://journals.plos.org/plosone/s/submission-guidelines#loc-laboratory-protocols. Additionally, PLOS ONE offers an option for publishing peer-reviewed Lab Protocol articles, which describe protocols hosted on protocols.io. Read more information on sharing protocols at https://plos.org/protocols?utm_medium=editorial-email&utm_source=authorletters&utm_campaign=protocols.

As the corresponding author, your ORCID iD is verified in the submission system and will appear in the published article. PLOS supports the use of ORCID, and we encourage all coauthors to register for an ORCID iD and use it as well. Please encourage your coauthors to verify their ORCID iD within the submission system before final acceptance, as unverified ORCID iDs will not appear in the published article. *Only*   the individual author can complete the verification step; PLOS staff *cannot*   verify ORCID iDs on behalf of authors.

We look forward to receiving your revised manuscript.

Kind regards,

Jordan Llego, PhD ELM, D. Hon. Ex., PhDN, RN

Academic Editor

PLOS One

Journal Requirements:

If the reviewer comments include a recommendation to cite specific previously published works, please review and evaluate these publications to determine whether they are relevant and should be cited. There is no requirement to cite these works unless the editor has indicated otherwise.

Additional Editor Comments:

Thank you for submitting the revised version of the manuscript. The revised paper demonstrates substantial improvement, reflecting significant effort to address the concerns raised during the previous review. The manuscript is now better organized, with strengthened analytical reporting, and key sections such as the discussion, conclusion, and limitations are more focused and aligned with the study’s objectives. Notably, the clarification of the study design, the more explicit reporting of structural equation modeling results, and the clearer articulation of the study’s theoretical and practical relevance are commendable.

However, following careful editorial assessment, the manuscript is not yet ready for acceptance in its current form. Although the remaining issues do not warrant rejection, they are sufficiently significant to require further revision before a final decision can be made. Most notably, there is an inconsistency in the ethics and consent documentation. The manuscript reports verbal informed consent, while other submission materials indicate written consent. This inconsistency must be corrected thoroughly and consistently across the manuscript, ethics statement, and all submission metadata. Additionally, the ethics approval should clearly support the form of consent that was actually obtained, including any waiver of written consent if applicable.

There are also unresolved concerns regarding the reporting and justification of the intolerance of uncertainty instrument. The manuscript utilizes the 27-item measure, yet some justification relies on discussion of the shorter IUS-12, which may cause confusion. The rationale, validation support, and interpretation should consistently correspond to the instrument actually administered. The reference list must also be reviewed carefully, as inclusion of any retracted source is unacceptable and such citations must be removed or replaced with valid references.

Although the discussion section has improved, some passages still contain wording that may be interpreted as causal or overly prescriptive. As the study is cross-sectional and based on self-report data collected at a single time point, interpretations must remain associative rather than causal throughout the manuscript. Statements suggesting that one construct improves, determines, or directly leads to another should be revised. Furthermore, the manuscript would benefit from a clearer statement regarding common method bias. Even if no formal statistical test was conducted, any procedural steps taken to reduce this concern should be briefly explained, and the issue should be acknowledged transparently in the limitations.

The manuscript is therefore invited for further revision. In preparing the next version, all remaining concerns should be addressed carefully, accurately, and consistently throughout the full manuscript and submission materials. The authors are encouraged to review the revised paper line by line to ensure that no conflicting statements remain and that all reporting is fully aligned with journal standards. A clear and detailed point-by-point response should also be provided, explaining exactly how each concern has been addressed.

[Note: HTML markup is below. Please do not edit.]

[NOTE: If reviewer comments were submitted as an attachment file, they will be attached to this email and accessible via the submission site. Please log into your account, locate the manuscript record, and check for the action link "View Attachments". If this link does not appear, there are no attachment files.]

To ensure your figures meet our technical requirements, please review our figure guidelines: https://journals.plos.org/plosone/s/figures

You may also use PLOS’s free figure tool, NAAS, to help you prepare publication quality figures: https://journals.plos.org/plosone/s/figures#loc-tools-for-figure-preparation.

NAAS will assess whether your figures meet our technical requirements by comparing each figure against our figure specifications.

# Author comment: Author response to Decision Letter 3

**DOI:** 10.1371/journal.pone.0350761.r006

12 May 2026

Dear Editor,

We thank you for your careful evaluation of our revised manuscript and for the constructive comments provided. We appreciate the opportunity to further revise and improve the manuscript.

In preparing this revision, we have carefully addressed all points raised in your editorial assessment. The manuscript has been reviewed in detail to ensure clarity, internal consistency, and alignment with the reporting standards of PLOS ONE. In particular, we have ensured consistency in ethical reporting and informed consent documentation across all sections and submission materials, revised the wording throughout the manuscript to avoid causal interpretations in accordance with the cross-sectional design, and strengthened methodological transparency by explicitly addressing common method bias.

All changes have been incorporated into the revised manuscript, and a detailed point-by-point response is provided below to indicate how each comment has been addressed.

We hope that the revised manuscript meets the journal’s requirements and is suitable for publication.

Additional Editor Comments:

1- Thank you for submitting the revised version of the manuscript. The revised paper demonstrates substantial improvement, reflecting significant effort to address the concerns raised during the previous review. The manuscript is now better organized, with strengthened analytical reporting, and key sections such as the discussion, conclusion, and limitations are more focused and aligned with the study’s objectives. Notably, the clarification of the study design, the more explicit reporting of structural equation modeling results, and the clearer articulation of the study’s theoretical and practical relevance are commendable.

However, following careful editorial assessment, the manuscript is not yet ready for acceptance in its current form. Although the remaining issues do not warrant rejection, they are sufficiently significant to require further revision before a final decision can be made. Most notably, there is an inconsistency in the ethics and consent documentation. The manuscript reports verbal informed consent, while other submission materials indicate written consent. This inconsistency must be corrected thoroughly and consistently across the manuscript, ethics statement, and all submission metadata. Additionally, the ethics approval should clearly support the form of consent that was actually obtained, including any waiver of written consent if applicable.

There are also unresolved concerns regarding the reporting and justification of the intolerance of uncertainty instrument. The manuscript utilizes the 27-item measure, yet some justification relies on discussion of the shorter IUS-12, which may cause confusion. The rationale, validation support, and interpretation should consistently correspond to the instrument actually administered. The reference list must also be reviewed carefully, as inclusion of any retracted source is unacceptable and such citations must be removed or replaced with valid references.

Response: We sincerely thank the Editor for identifying this important inconsistency in the ethics and consent documentation. We have carefully reviewed the manuscript and the submission materials and confirm that verbal informed consent was obtained from all participants. The previous statement indicating that participants signed a written consent form was an error in wording in the Ethics Statement and did not reflect the actual consent procedure used in the study.

To correct this issue, we have revised the Ethics Statement and all relevant parts of the manuscript to consistently state that verbal informed consent was obtained. We also clarified that the use of verbal informed consent, including the waiver of written informed consent, was approved by the Ethics Committee of Iran University of Medical Sciences under the ethics code IR.IUMS.REC.1401.561.

The revised Ethics Statement now reads:

“This study was conducted with the approval of the Ethics Committee of Iran University of Medical Sciences under the ethics code IR.IUMS.REC.1401.561. Prior to data collection, the study objectives were explained to all participants, and verbal informed consent was obtained voluntarily from each participant without any coercion. The use of verbal informed consent and the waiver of written informed consent were approved by the Ethics Committee. All participant information was kept strictly confidential by the researchers.”

We have also reviewed the submission metadata and accompanying documents to ensure consistency regarding the consent procedure.

2- Although the discussion section has improved, some passages still contain wording that may be interpreted as causal or overly prescriptive. As the study is cross-sectional and based on self-report data collected at a single time point, interpretations must remain associative rather than causal throughout the manuscript. Statements suggesting that one construct improves, determines, or directly leads to another should be revised. Furthermore, the manuscript would benefit from a clearer statement regarding common method bias. Even if no formal statistical test was conducted, any procedural steps taken to reduce this concern should be briefly explained, and the issue should be acknowledged transparently in the limitations.

Response: We sincerely thank the Editor for this important and insightful comment. We fully agree that, given the cross-sectional design and reliance on self-report data collected at a single time point, all interpretations should remain strictly associative rather than causal.

In response, we conducted a comprehensive and systematic revision of the manuscript to eliminate potentially causal language. Specifically, terms such as “effect,” “impact,” “influence,” and “improves” were carefully reviewed and replaced with more appropriate expressions such as “association,” “relationship,” and “is related to” throughout the Abstract, Results, Discussion, and Conclusion sections. This revision ensures full consistency with the observational nature of the study.

In addition, we have further clarified the interpretation of findings in the Discussion section by explicitly framing the results in terms of associations rather than causal mechanisms. The Conclusion has also been revised to avoid prescriptive or causal implications

Regarding common method bias, we have now addressed this issue explicitly. A statement has been added to the Methods section describing procedural steps taken to reduce this bias, including ensuring participant anonymity, emphasizing voluntary participation, and using standardized and validated instruments. Furthermore, the Limitations section has been expanded to transparently acknowledge the potential influence of common method bias due to the use of self-report data collected at a single time point.

We believe that these revisions have substantially improved the interpretive rigor and methodological transparency of the manuscript, and we hope that they fully address the Editor’s concern.

3- The manuscript is therefore invited for further revision. In preparing the next version, all remaining concerns should be addressed carefully, accurately, and consistently throughout the full manuscript and submission materials. The authors are encouraged to review the revised paper line by line to ensure that no conflicting statements remain and that all reporting is fully aligned with journal standards. A clear and detailed point-by-point response should also be provided, explaining exactly how each concern has been addressed.

Response: We sincerely thank the Editor for inviting us to revise the manuscript further and for providing clear guidance for the next revision. We have carefully reviewed the entire manuscript line by line to ensure that all remaining concerns have been addressed accurately and consistently.

In particular, we checked the manuscript and submission materials for consistency in ethical reporting and informed consent documentation. All references to informed consent have now been revised to consistently indicate that verbal informed consent was obtained, as approved by the Ethics Committee of Iran University of Medical Sciences, and that the requirement for written informed consent was waived.

We also systematically reviewed the manuscript to ensure that the interpretation of findings remains appropriate for a cross-sectional, self-report study. Potentially causal or overly prescriptive wording was revised throughout the Abstract, Results, Discussion, Conclusion, and Practical Implications sections.

In addition, we added a clearer statement regarding common method bias in the Methods section and expanded the Limitations section to acknowledge this issue transparently.

A detailed point-by-point response has been provided below, explaining exactly how each concern has been addressed. We hope that the revised manuscript and accompanying response letter now fully meet the journal’s reporting standards.

Attachment Submitted filename: Our_reply_to_reviewers_auresp_3.docx

# Editor report: Decision Letter 3

**DOI:** 10.1371/journal.pone.0350761.r007

**Authors:** Jordan Llego

18 May 2026

Modeling the Effect of Psychological Capital on Nurses' Job Performance with the Moderating Role of Intolerance of Uncertainty

PONE-D-25-35558R3

Dear Dr. Ebrahimi,

We’re pleased to inform you that your manuscript has been judged scientifically suitable for publication and will be formally accepted for publication once it meets all outstanding technical requirements.

Within one week, you’ll receive an e-mail detailing the required amendments. When these have been addressed, you’ll receive a formal acceptance letter and your manuscript will be scheduled for publication.

An invoice will be generated when your article is formally accepted. Please note, if your institution has a publishing partnership with PLOS and your article meets the relevant criteria, all or part of your publication costs will be covered. Please make sure your user information is up-to-date by logging into Editorial Manager at Editorial Manager® and clicking the ‘Update My Information' link at the top of the page. For questions related to billing, please contact billing support.

If your institution or institutions have a press office, please notify them about your upcoming paper to help maximize its impact. If they’ll be preparing press materials, please inform our press team as soon as possible -- no later than 48 hours after receiving the formal acceptance. Your manuscript will remain under strict press embargo until 2 pm Eastern Time on the date of publication. For more information, please contact onepress@plos.org.

Kind regards,

Jordan Llego, PhD ELM, D. Hon. Ex., PhDN, RN

Academic Editor

PLOS One

Additional Editor Comments (optional):

Thank you for submitting the revised manuscript. The authors have addressed the major concerns from the previous editorial assessment, including ethical reporting, informed consent documentation, clarification of the intolerance of uncertainty instrument, reduction of causal language, and acknowledgment of common method bias. The manuscript has improved significantly and is now suitable for acceptance.

Before production, the manuscript should receive minor editorial and proofreading corrections to address typographical, formatting, and language issues. This includes correcting spelling errors in tables, ensuring consistent terminology, and using terms like “effect” cautiously given the cross-sectional design. These are editorial matters and do not require further scientific revision.

Minor points to flag for production/proofreading:

1. Change “Tell” to “Tel.” in the corresponding author information.

2. Correct table errors such as “Cofficent” to “Coefficient” and “T-Statictic” to “T-statistic.”

3. Replace “Causal relations” in Table 4 with “Hypothesized relationships” or “Structural paths.”

4. Consider changing the title wording from “Effect” to “Association” or “Relationship” to better match the cross-sectional design.

5. Remove or soften remaining causal/prescriptive wording such as “effect,” “enhance,” “improving,” or “influence” where needed.

6. Review reference formatting and minor language issues before publication.

Reviewers' comments:

# Editor report: Acceptance letter

**DOI:** 10.1371/journal.pone.0350761.r008

**Authors:** Jordan Llego

PONE-D-25-35558R3

PLOS One

Dear Dr. Ebrahimi,

I'm pleased to inform you that your manuscript has been deemed suitable for publication in PLOS One. Congratulations! Your manuscript is now being handed over to our production team.

At this stage, our production department will prepare your paper for publication. This includes ensuring the following:

* All references, tables, and figures are properly cited

* All relevant supporting information is included in the manuscript submission,

* There are no issues that prevent the paper from being properly typeset

You will receive further instructions from the production team, including instructions on how to review your proof when it is ready. Please keep in mind that we are working through a large volume of accepted articles, so please give us a few days to review your paper and let you know the next and final steps.

Lastly, if your institution or institutions have a press office, please let them know about your upcoming paper now to help maximize its impact. If they'll be preparing press materials, please inform our press team within the next 48 hours. Your manuscript will remain under strict press embargo until 2 pm Eastern Time on the date of publication. For more information, please contact onepress@plos.org.

You will receive an invoice from PLOS for your publication fee after your manuscript has reached the completed accept phase. If you receive an email requesting payment before acceptance or for any other service, this may be a phishing scheme. Learn how to identify phishing emails and protect your accounts at https://explore.plos.org/phishing.

If we can help with anything else, please email us at customercare@plos.org.

Thank you for submitting your work to PLOS ONE and supporting open access.

Kind regards,

PLOS ONE Editorial Office Staff

on behalf of

Dr. Jordan Llego

Academic Editor

PLOS One
